# Cisplatin Enhances Hepatitis B Virus Replication and PGC-1α Expression through Endoplasmic Reticulum Stress

**DOI:** 10.1038/s41598-018-21847-3

**Published:** 2018-02-22

**Authors:** Xiaosong Li, E. Pan, Junke Zhu, Lei Xu, Xuemei Chen, Jingjing Li, Li Liang, Yuan Hu, Jie Xia, Juan Chen, Wannan Chen, Jieli Hu, Kai Wang, Ni Tang, Ailong Huang

**Affiliations:** 1 Key Laboratory of Molecular Biology for Infectious Diseases (Ministry of Education), Institute for Viral Hepatitis, Department of Infectious Diseases, The Second Affiliated Hospital, Chongqing Medical University, Chongqing, China; 2 Key Laboratory of Tumor Microbiology, Fujian Medical University, Fuzhou Fujian, China; 3 The Collaborative Innovation Center for Diagnosis and Treatment of Infectious Diseases (CCID), Zhejiang University, Hangzhou, China

## Abstract

Chronic hepatitis B infection remains a serious public health issue worldwide. Hepatitis B virus (HBV) reactivation is commonly reported in patients receiving anticancer therapy, immunosuppressive therapy, or organ and tissue transplantation. However, the precise mechanisms underlying chemotherapeutic agent-related HBV reactivation remain unclear. Here, we report that peroxisome proliferator-activated receptor gamma coactivator 1 alpha (PGC-1α) plays a central role in cisplatin-induced HBV transcription and replication. First, cisplatin treatment upregulated the expression levels of PGC-1α and hepatocyte nuclear factor 4 alpha (HNF-4α) in both HBV-replicating cells and an HBV-transgenic mouse model. PGC-1α coactivates with HNF-4α, which interacts with a core promoter and enhancer II region of HBV genome, thereby promoting HBV production. In contrast, knockdown of PGC-1α and HNF-4α by RNA interference in hepatoma cells reversed HBV activation in response to cisplatin. Additionally, PGC-1α upregulation depended on cisplatin-mediated endoplasmic reticulum (ER) stress. We further observed that the recruitment of cyclic AMP-responsive element-binding protein plays a crucial role for PGC-1α transcriptional activation in cisplatin-treated cells. Finally, pharmacologic inhibition of ER stress impaired PGC-1α upregulation and HBV production induced by cisplatin treatment. These findings demonstrate novel molecular mechanisms indicating that ER stress-PGC1α signaling pathway plays a critical role in cisplatin-evoked HBV reactivation.

## Introduction

Hepatitis B virus (HBV) infection has become a serious global public health issue. It is estimated that approximately 350 million people worldwide have chronic hepatitis B infection^1^. HBV is a partially double-stranded, circular 3.2 kb DNA that belongs to the orthohepadnavirus of the Hepadnaviridae family that infects hepatocytes and leads to liver pathologic changes. The HBV genome contains four overlapping open reading frames that encode HBsAg, HBeAg, HBV polymerase, and HBx protein^2^. After infection of hepatocytes, the HBV nucleocapsids are released into the cytoplasm, and the relaxed circular DNA (rcDNA) is converted into a covalently closed circular DNA (cccDNA), which is maintained in the nucleus and served as template for the transcription of all viral genes. Viral replication occurs via 3.5 kb transcript (pregenomic RNA) by reverse transcription process. Matured rcDNA-containing viral capsids can be secreted via interaction with the envelope proteins as progeny virions or can re-deliver the rc-DNA to the nucleus to build up the cccDNA pool^3^.

Hepatitis B virus (HBV) reactivation is a potentially serious disorder that was first reported in the mid-1970s in patients who underwent cytotoxic drug therapy and kidney transplantation^4,5^. HBV reactivation is defined as a sudden increase in serum HBV DNA (>10-fold increase from the baseline) and serum alanine aminotransferase (ALT) level elevation (two-three-fold elevation above the baseline) associated with hepatitis flare^6^. HBV reactivation frequency in chronic hepatitis B patients undergoing chemotherapy ranges from 4 to 68%, depending on the chemotherapeutic agents, chemotherapy regimen, and HBV serological status^7^. HBV reactivation can interrupt chemotherapy and induce hepatic injury or severe liver failure.

The endoplasmic reticulum (ER) is a cellular organelle involved in lipid metabolism, steroid hormone production, and intracellular calcium storage^8^. Conditions such as viral infection, energy deprivation, hypoxia, or exposure to excessive oxidative stress can trigger ER stress or the unfolded protein response (UPR). ER stress can initiate unfolded protein response (UPR) via three different sensors: inositol-requiring enzyme 1 (IRE1), activating transcription factor 6 (ATF6), and protein kinase R-like endoplasmic reticulum kinase (PERK). Recent reports indicated that ER stress is involved in both cisplatin-induced tumour cell death and drug resistance, depending on the concentration of cisplatin^9^. More recently, chronic ER stress due to HBV infection has been reported to induce oxidative DNA lesions, mutagenesis, and cellular inflammation, thereby being involved in the pathogenesis of viral-related liver injury^10^. Mutation in PreS1/PreS2 region or HBV genotype G infection, which is associated with impaired secretion of HBsAg, induced ER stress and increased ROI levels in HBV-expressing cells^11^. In an HBV-transgenic mouse model, intracellular retention of the HBV surface antigen (HBsAg) in hepatocytes induced ER stress and caused “ground-glass” morphologic changes and hypersensitivity to cytokines^12^.

Cisplatin is an effective chemotherapeutic agent often used to treat various types of cancers, including lymphomas, sarcomas, ovarian cancer, cervical cancer, small cell lung cancer, bladder cancer, and others^13^. However, the risk of HBV reactivation in cancer patients receiving chemotherapy increases. Several clinical studies have reported that HBV reactivation occurs in patients with HCC undergoing chemotherapy by using cisplatin and epirubicin^14,15^. The precise mechanisms in HBV reactivation are unknown. Impairment of host immune system over viral replication is thought to be the initial factor. However, nearly half of HBV reactivation occurred in the early stage of chemotherapeutic regimen^16^, indicating that mechanisms other than perturbation of the balance between immunity and viral replication, such as direct stimulatory effects of anticancer drugs, may be partially responsible for HBV reactivation.

An increasing amount of data suggested that cytotoxic chemotherapy, including doxorubicin, etoposide, or vincristine, may directly increase HBV DNA and HBsAg secretion in a dose-dependent manner^17,18^. However, an in-depth exploration of the molecular mechanisms involving chemotherapeutic agent- or immunosuppression-related HBV reactivation is still missing.

Here, we investigated the role of cisplatin in HBV replication regulation. We found that cisplatin administration dramatically enhanced HBV DNA production in HBV replicative cells and in a mouse model through ER stress-peroxisome proliferator-activated receptor gamma coactivator 1 alpha (PGC-1α) signalling pathway activation. This study aimed to broaden the knowledge on biological processes underlying persistent HBV infection and HBV reactivation.

## Results

### Cisplatin directly upregulates HBV replication in HBV-expressing liver cells

**Figure 1:**
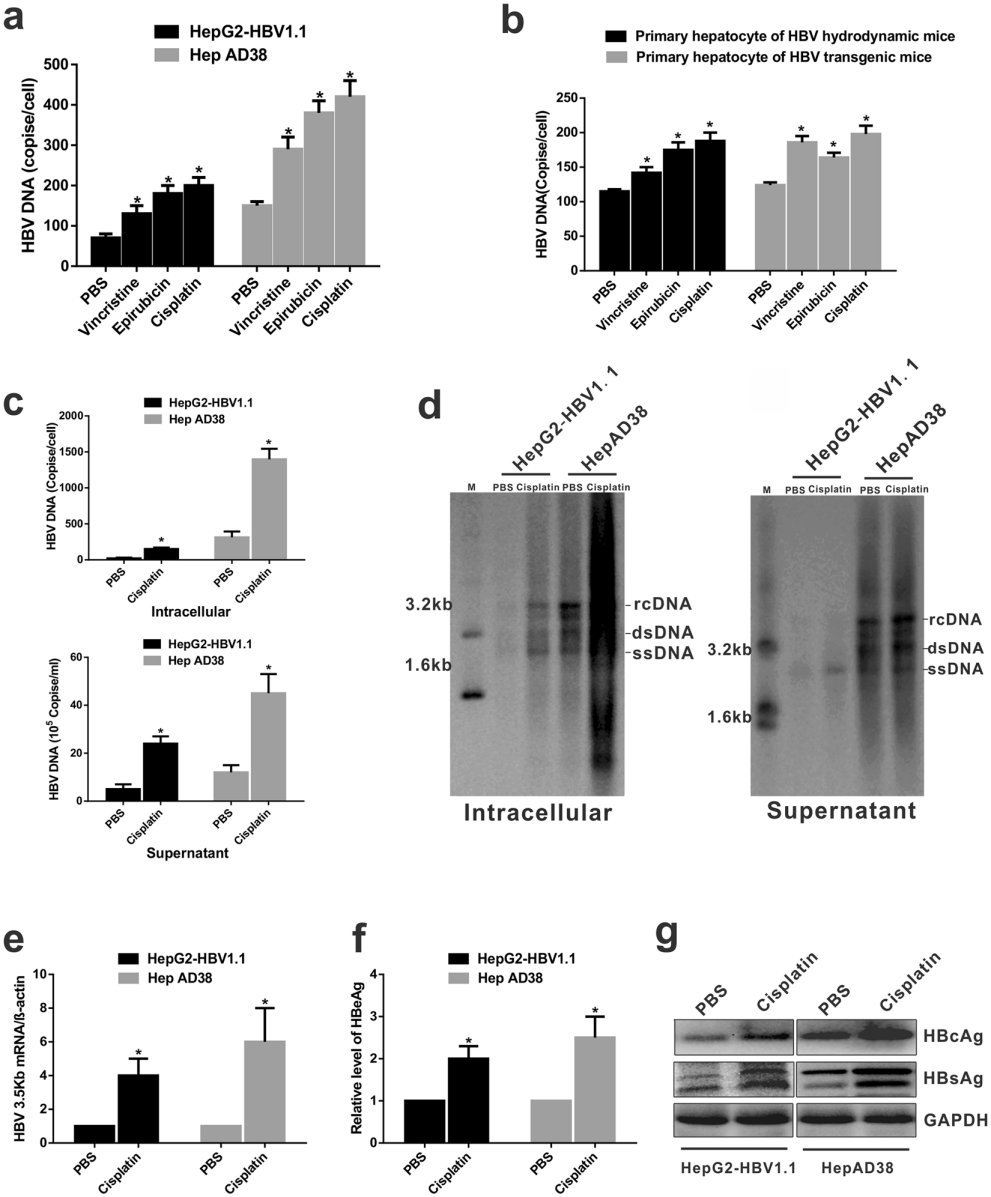
Cisplatin promotes HBV replication in HBV-expressing hepatoma cells. (**a**) Chemotherapy treatment-upregulated HBV replication in stable HBV-expressing cell lines HepAD38 and HepG2-HBV1.1. Cells were treated with 6 μM cisplatin, 0.3 μM vincristine, or 0.6 μM epirubicin for 24 h. Intracellular HBV DNA was extracted and quantified by quantitative PCR analysis. Values were normalized to PBS control. The data represent the mean value from independent experiments (n = 3). *p < 0.05 vs. PBS control. (**b**) Primary hepatocytes were isolated from HBV transgenic mice (HBV-Tg mice) or HBV hydrodynamic mice. Cells were treated with the indicated concentration of the chemotherapy drug. Intracellular HBV DNA was quantified as described in **a**. (**c**) HepAD38 and HepG2-HBV1.1 cells were treated with 6 μM cisplatin. Cell lysates and culture supernatants were harvested at day 4 after treatment. Intracellular (top panel) and supernatant (bottom panel) HBV DNA levels were quantified by real-time PCR assay. Data represent the means $\pm$ SD (independent experiments, n = 3); *p < 0.05 vs. PBS control. (**d**) Intracellular or secreted HBV DNA was determined by Southern blot analysis. rc DNA, relaxed circular DNA; ds DNA, double-stranded DNA; ss DNA, single-stranded DNA. (**e**,**f**) Quantification of HBV 3.5 kb mRNA levels (**e**) or secreted HBeAg (f) in HepAD38 and HepG2-HBV1.1 cells. Data represent the mean value from independent experiments (n = 3). *p < 0.05 vs. PBS control. (**g**) Intracellular HBcAg and HBsAg were measured by western blotting. GAPDH was used as loading control. The cropped blots are used in the figure and full length blots are presented in Supplementary Fig. S7.

We previously found that epirubicin and vincristine directly promoted HBV replication in stable HBV-expressing hepatoma cells^19,20^. In this study, we first explored whether other cytotoxic chemotherapy agents could directly upregulate HBV replication *in vitro*, in the context of hepatoma or hepatocytes expressing HBV without an innate and adaptive immune system. Stable HBV-expressing cell line HepAD38 and HepG2-HBV1.1 cells were treated with the optimal dose of chemotherapy agents (Supplementary Fig. 1a). We found that cytotoxic chemotherapy induces a sharp increase in HBV DNA levels (Fig. 1a). Primary hepatocytes, derived from an HBV replication mouse model including transgenic mice and hydrodynamic injection mouse model, were used to examine the effects of cytotoxic agents on HBV replication. An approximately 10–40-fold induction of HBV DNA levels was observed in primary hepatocytes derived from HBV-Tg mice and hydrodynamic mouse model after treatment with chemotherapy agents for 5 days. Among the cytotoxic agents, cisplatin displayed the strongest stimulatory effects (Fig. 1b). Consequently, cisplatin was chosen as the representative chemotherapy agent for the experiments. Treatment with 6 μM cisplatin significantly increased both intracellular and secreted HBV DNA levels (Fig. 1c), which was confirmed by Southern blot analysis (Fig. 1d). Moreover, cisplatin treatment enhanced HBV replicative intermediates and increased HBV 3.5 kb mRNA, intracellular HBsAg and HBcAg expression, and HBeAg levels in the culture supernatants (Fig. 1e–g). In addition, similar enhanced HBV replication was examined by cisplatin treatment in HBV-infected HepG2-NTCP cells (Supplementary Fig. 1b,c). When cells were pretreated with nucleotide analogue inhibitors, including lamivudine and entecavir, cisplatin-mediated upregulation of HBV replication was not impaired when comparing PBS treatments (Supplementary Fig. 1d,e). The aforementioned results suggested that chemotherapy agents, especially cisplatin, strengthen HBV replication and transcription in HBV-expressing liver cells.

### Cisplatin treatment enhances HBV replication in the HBV mouse model

**Figure 2:**
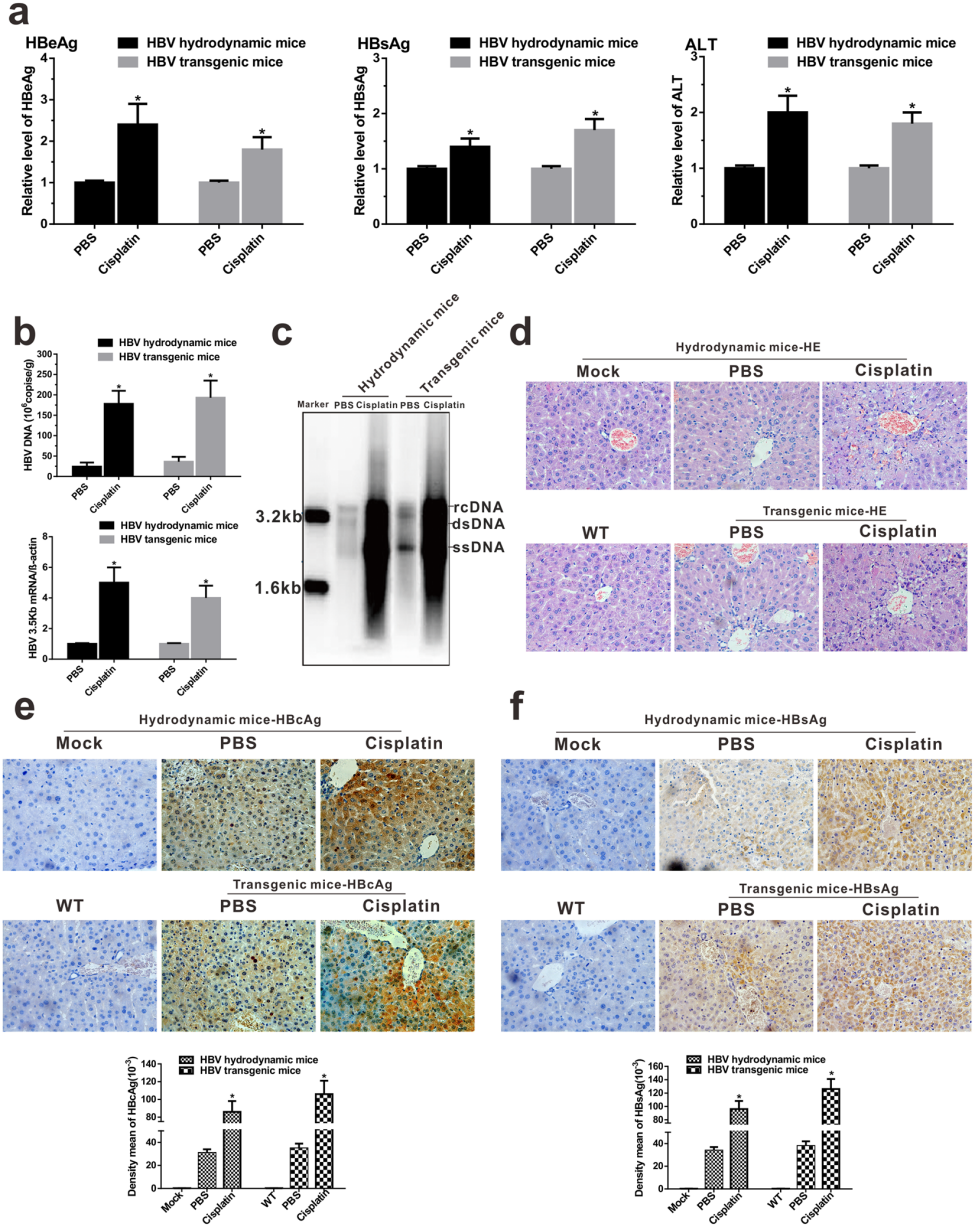
Cisplatin administration enhances viral biosynthesis in HBV transgenic and HBV hydrodynamic mouse model. (**a**) Serological markers of HBeAg, HBsAg, and ALT were assayed (n = 6/group). (**b**) HBV DNA and HBV 3.5 kb mRNA in the liver was detected by real-time PCR. *p < 0.05. (**c**) HBV replication intermediates in the liver were detected by Southern blotting. (**d** to **f**) Liver tissue sections were H&E stained (**d**, magnification, ×200) or stained with anti-HBc antibodies and anti-HBs antibody (**e** and **f**, respectively). Mock, mock-treated group; WT, wild-type C57BL/6 J mouse. IHC staining was quantitatively analyzed using Image-Pro Plus software; *p < 0.05.

We investigated whether cisplatin could strengthen HBV replication in the HBV mouse model. Six HBV-Tg mice or mice that received HI of pHBV1.3 were treated with 6 mg/kg cisplatin or phosphate-buffered saline (control) for 5 days. We found that cisplatin administration significantly increased serum HBsAg, HBeAg, and HBV DNA levels by approximately 1.5-, 2.5-, and 30-fold, respectively, in the hydrodynamic HBV mouse model (Fig. 2a,b; Supplementary Fig. 2a). Consistently, HBV DNA levels, HBV replication intermediates in the liver, HBV 3.5 kb mRNA, and HBsAg and HBcAg in hepatocytes were markedly enhanced after cisplatin administration (Fig. 2c–f; Supplementary Fig. 2b,c). Similarly, HBV replication enhancement was observed in cisplatin-treated HBV-Tg mice (Fig. 2). Previous studies reported that HBV reactivation with chemotherapy usually evokes moderate or severe liver damage in patients^14,15^. To determine whether cisplatin treatment could induce liver injury, we assessed serum ALT activity levels and histopathological lesions after cisplatin treatment. Serum ALT was elevated by approximately 2- and 1.8-fold in the HI mouse model and HBV-Tg mouse model, respectively (Fig. 2a). An apparent inflammatory cell infiltration in the portal area was easily observed in the cisplatin-treated HBV mouse model (Fig. 2d). These results indicated that cisplatin treatment augments HBV DNA replication in the HBV mouse model.

### Cisplatin augments viral HBV transcription through HNF-4α and PGC-1α activation

**Figure 3:**
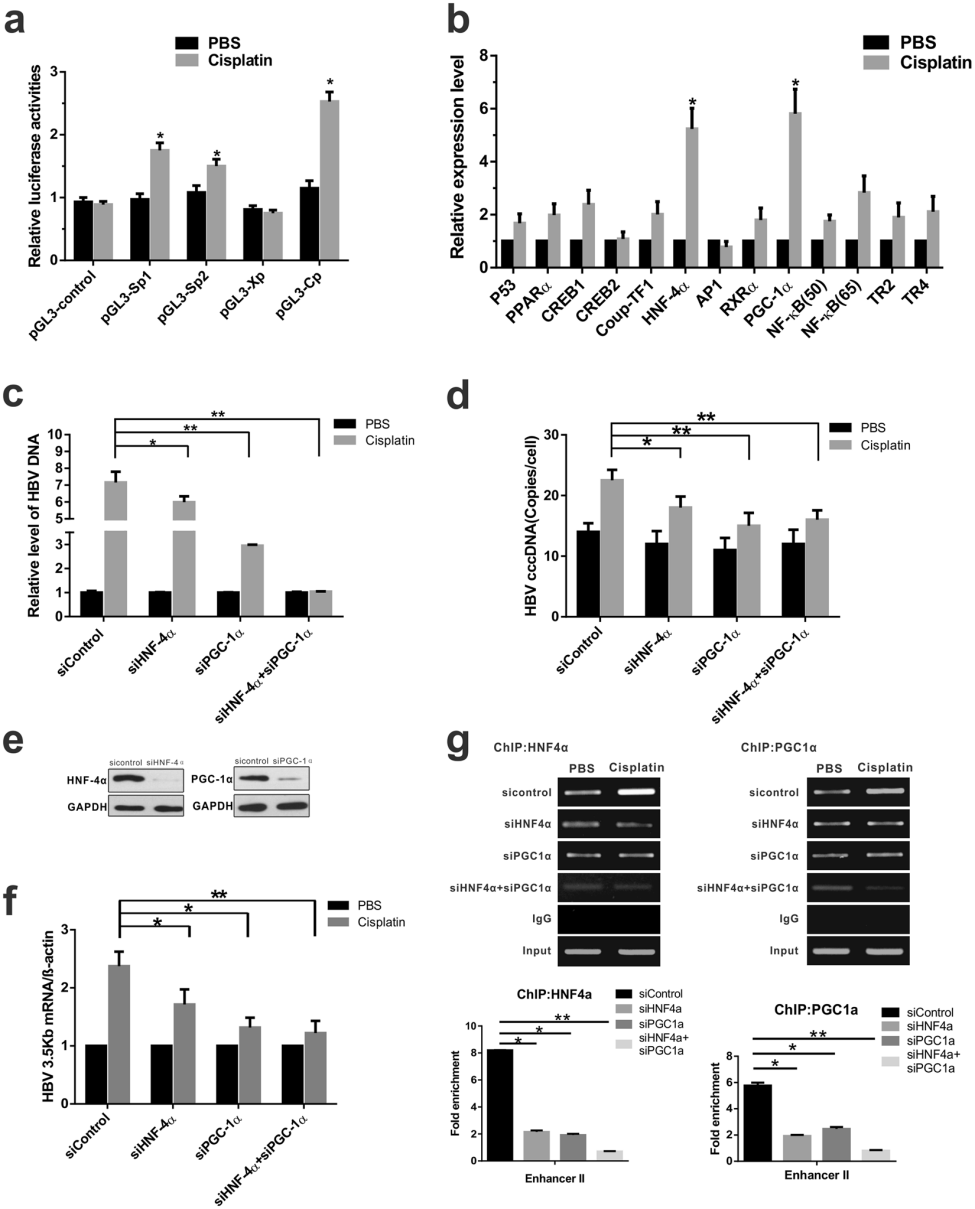
HNF4α and PGC1α are upregulated by cisplatin treatment and involved in HBV reactivation in HBV-expressing cells. (**a**) HepG2 cells were transfected with the luciferase reporter vectors containing HBV core, PreS1, PreS2, or X promoter. After 12 h of transfection, cells were treated with 1.3 μg/ml cisplatin for 24 h. Data represent the means $\pm$ SD (independent experiments, n = 3); *p < 0.05 vs. vector control. (**b**) mRNA levels of HNF4α and PGC1α expression in HepAD38 cells. *p < 0.05 vs. PBS control. (**c** to **d**) Quantification of HBV DNA and cccDNA in HepAD38 cells. Data are mean $\pm$ SD (independent experiments, n = 3); *p < 0.05, **p < 0.01. (**e**) ShRNA-mediated knockdown efficiency of HNF-4α and PGC-1α were verified by Western blot analysis. (**f**) Quantification of HBV 3.5 kb mRNA in HepAD38 cells. Data are mean $\pm$ SD (independent experiments, n = 3); *p < 0.05, **p < 0.01. (**g**) Chromatin immunoprecipitation was performed using anti-HNF4α, anti-PGC1α antibody, or control IgG. The immunoprecipitated chromatin was amplified by primers spanning the HNFα-binding site in the core promoter region of HBV genome. *p < 0.05, **p < 0.01 vs. siControl.

We investigated the mechanism in HBV replication due to cisplatin administration. We found that cisplatin treatment significantly increased the core promoter activity by approximately three-fold and activated preS1 and preS2 promoters activity with much less efficiency (Fig. 3a). Host factors, especially numerous transcription factors, can stimulate or suppress HBV genome transcription and replication. To identify the transcription factors involved in HBV activation by cisplatin, a real-time PCR assay was performed. The mRNA and protein levels of HNF-4α and PGC-1α were robustly upregulated in HBV-expressing cells following cisplatin treatment (Fig. 3b, Supplementary Fig. 3a).

**Figure 4:**
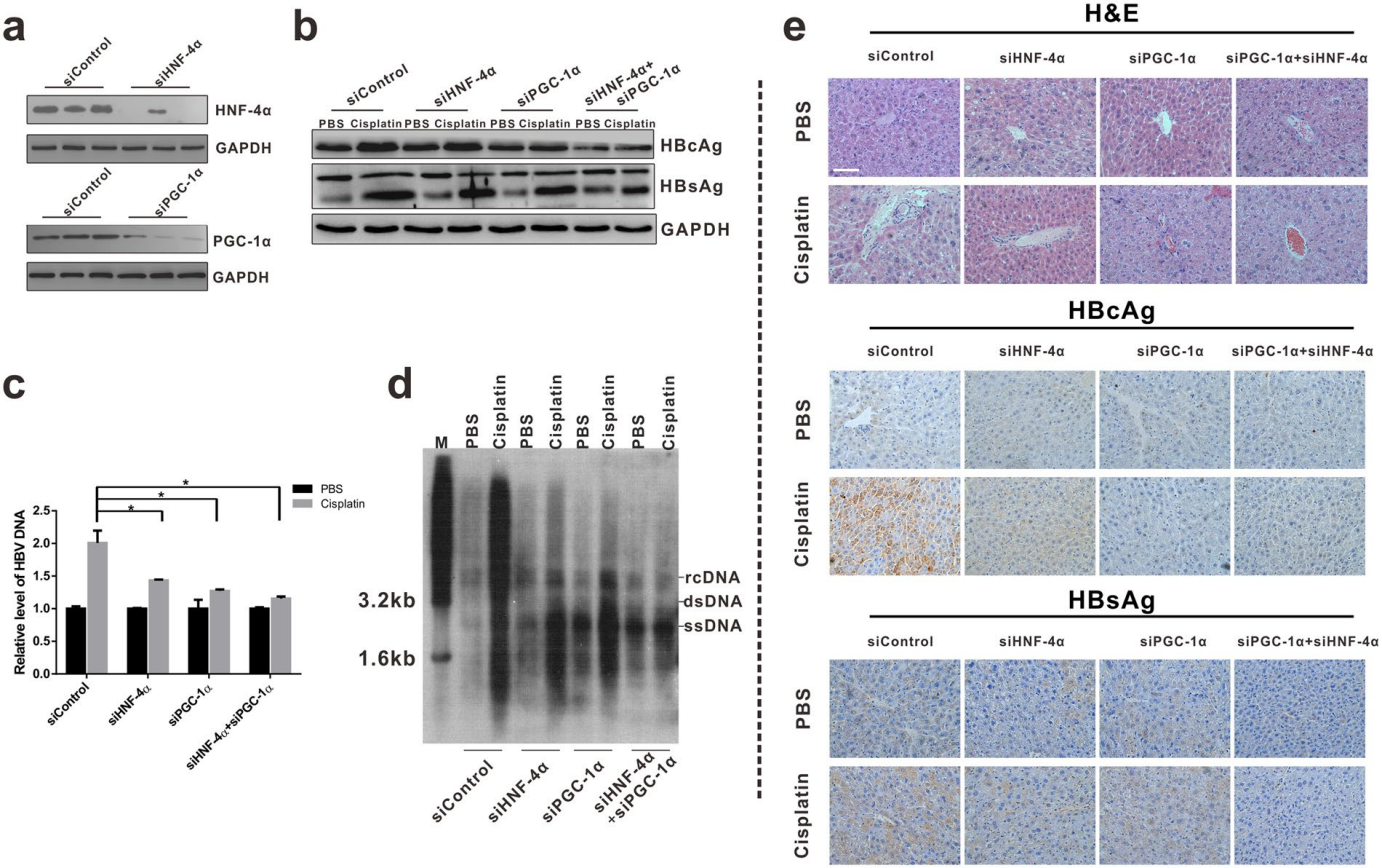
HNF4α and PGC1α ablation reduces cisplatin-induced HBV reactivation in HBV transgenic mouse model. The HBV transgenic mice (Background C57 BL/6 J) were randomly divided into eight groups (n = 6 for each group). Mice received 6.0 mg/kg cisplatin or PBS control daily for 5 days. On the sixth day, the mice were sacrificed and serum and tissue samples were collected. For siRNA experiments, adenoviruses AdR-siHNF4α, AdR-siPGC1α alone, or AdR-siHNF4α + AdR-siPGC1α were administered via the tail vein 24 h prior to cisplatin administration. (**a**) ShRNA-mediated knockdown efficiency of HNF-4α and PGC-1α were verified by Western blot analysis. (**b**) The representative expression levels of HBsAg and HBcAg in the mice liver were shown using western blot. (**c**) The sera of mice were collected and quantified for HBV DNA. *p < 0.05 (n = 6/group). (**d**) HBV replication intermediates in the liver were detected by Southern blotting. The cropped blots are used in the figure and full length blots are presented in Supplementary Fig. S8. (**e**) Representative H&E staining or immunohistochemical detection of HBsAg and HBcAg in liver tissues (magnification, ×200).

Subsequently, HNF-4α and PGC-1α was depleted via shRNA treatment, and the effects of cisplatin on viral DNA replication *in vitro* and in HBV-Tg mice were examined. As anticipated, induction of viral DNA replication following cisplatin treatment was strikingly reduced by treatment with siPGC-1α or siHNF-4α alone, with stronger inhibitory efficacy in PGC-1α-depleted cells. Combined treatment using siHNF-4α and siPGC-1α has the most inhibitory effects (Fig. 3c–f), indicating that HNF-4α and PGC-1α are vital for cisplatin-stimulated viral DNA replication. Moreover, knockdown of HNF-4α and PGC-1α similarly abolished cisplatin-mediated HBV transcription and viral protein production in HBV-Tg mouse (Fig. 4a–e).

HBV transcription is regulated by HNF-4α and the transcription coactivator PGC-1α, which binds to nuclear receptor response elements mainly found in its two enhancers^21^ (Supplementary Fig. 3b). Thus, whether PGC-1α and HNF-4α are directly responsible for cisplatin-enhanced viral transcription was addressed. Chromatin immunoprecipitation experiments were performed using antibodies against HNF-4α and PGC-1α, and cisplatin treatment was found to enhance recruitment of HNF-4α and PGC-1α to the HNF-4α binding site on Enh II (Supplementary Fig. 3c,d). However, silencing of HNF-4α and PGC-1α robustly impaired the binding capacity when siRNA was introduced to HBV-expressing cells (Fig. 3g). Generally, these results strongly indicated that cisplatin is a positive regulator for endogenous HNF-4α and PGC-1α expression, and HNF-4α and coactivator PGC-1α activation on the core promoter plays a significant role in cisplatin-triggered HBV synthesis.

### ER stress is required for cisplatin-induced HBV transcription

Previous studies showed that cisplatin treatment induces ER stress in hepatoma cells, and ER stress can activate cyclic AMP (cAMP)-responsive element-binding protein (CREB), which modulates PGC-1α transcription^22–24^. Our results showed that knockdown of PGC-1α demonstrated more inhibitory effects on cisplatin-stimulated HBV synthesis. Based on our results, we hypothesized that cisplatin-induced ER stress may mediate PGC-1α upregulation in a CREB-dependent manner.

**Figure 5:**
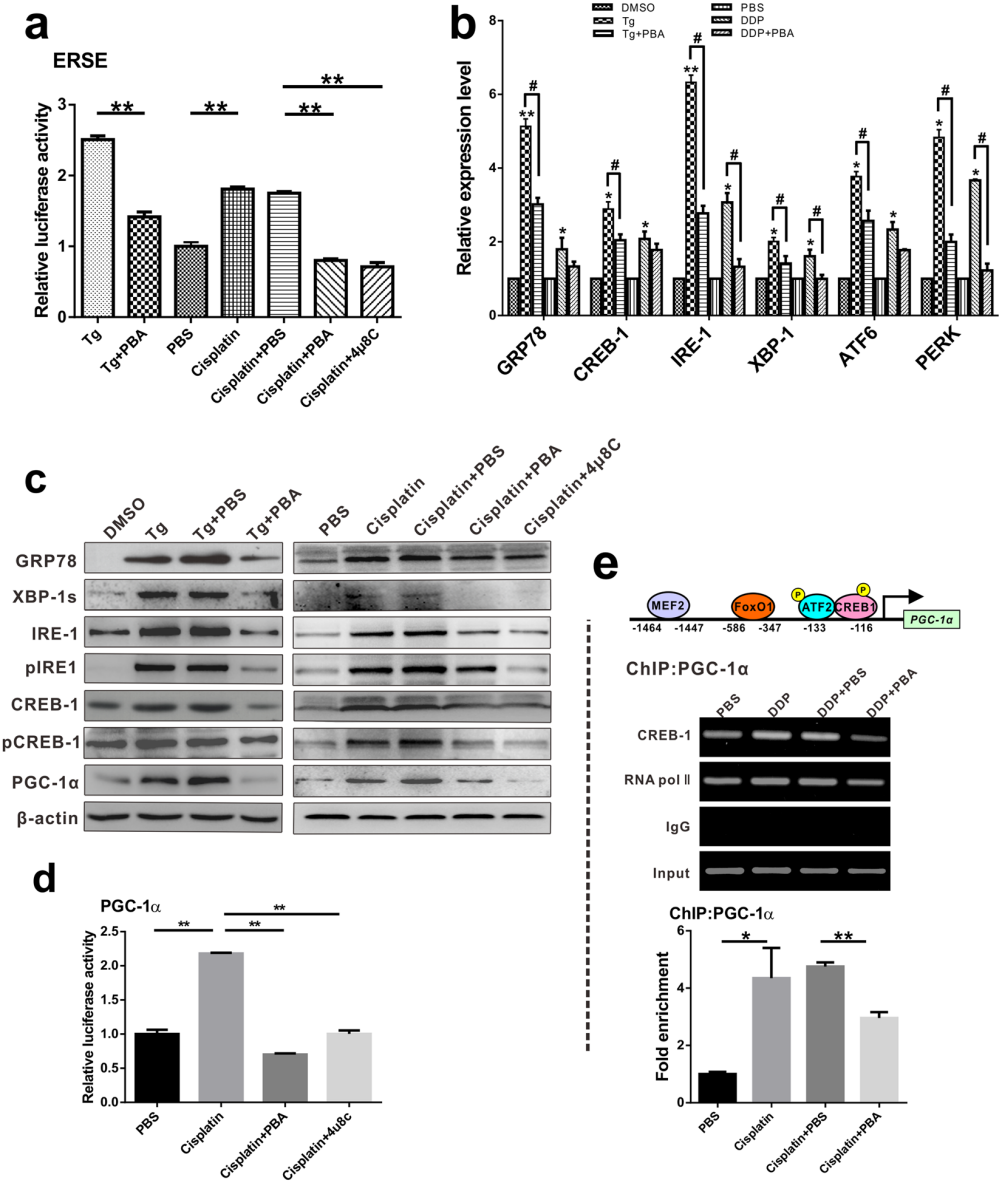
Cisplatin augments PGC1α expression through ER stress in HepAD38 cells. (**a**) The luciferase activity of ER stress response elements in HepAD38 cells. **p < 0.01. (**b**) The levels of the indicated ER inducible genes were determined by quantitative real-time PCR. HepAD38 cells were treated with cisplatin alone or cisplatin combined with 2.46 μg/ml ER stress inhibitor (4-PBA). Data are shown as mean $\pm$ SD (independent experiments, n = 3); *p < 0.05, **p < 0.01 vs PBS control, ^#^*p* < 0.05. (**c**) HepAD38 cells were treated as described in (**b**) or treated with cisplatin combined with 0.5 μg/ml IRE inhibitor (4 μ8C). Protein levels of ER stress genes were analyzed by western blot. The cropped blots are used in the figure and full length blots are presented in Supplementary Fig. S9. (**d**) PGC1α promoter activities in HepAD38 cells. Data represent the means $\pm$ SD (independent experiments, n = 3); **p < 0.01. (**e**) Chromatin immunoprecipitation assay of extracts from HepAD38 cells. Schematic representation of the PGC1α promoter with the MEF2, FoxO1, ATF2 and CREB1 binding sites. Quantitative PCR results are shown as mean $\pm$ SD (independent experiments, n = 3); *p < 0.05, **p < 0.01. RNA pol II recruited on PGC1α promoter were used as ChIP positive control.

First, the effects of cisplatin treatment were tested on ER stress response element-mediated transcriptional activity. Treatment with thapsigargin (Tg), an ER stress inducer, increased pERSE luciferase signalling in HepAD38 cells by approximately 2.5-fold compared with PBS control. Cisplatin treatment increased pERSE luciferase signalling by 1.5-fold compared with that in untreated cells (Fig. 5a). ER stress can activate UPR through three main signalling arms: IRE1, ATF6, and PERK. We accordingly examined which of these arms were activated in HepAD38 or HepG2.2.15 cells by cisplatin treatment and found that cisplatin increased the mRNA levels of phosphorylated IRE1, PERK, and ATF6 pathways (Fig. 5b; Supplementary Fig. 4a). Similarly, protein levels of spliced XBP-1s, CREB-1, and GRP78/BiP were robustly upregulated (2-, 2.5-, and 5-fold in HepAD38 and 2.2-, 1.8-, and 7.5-fold in HepG2.2.15 cells, respectively, compared to the PBS control) (Fig. 5c; Supplementary Fig. 4b,c). These data indicated that cisplatin activates each of the UPR branches, and phosphor-CREB protein was robustly elevated by cisplatin and Tg treatment, along with the increased protein and mRNA levels of PGC-1α. Therefore, cisplatin-induced PGC-1α upregulation by CREB phosphorylation is ER stress dependent.

We further validated whether ER stress inhibition can reverse cisplatin-induced upregulation of PGC-1α. 4-PBA and specific IRE1 inhibitor (4 μ8C)^25^ were used as ER stress inhibitors. Cisplatin-induced activation of ER stress markers was markedly reduced by PBA and 4 μ8C. Furthermore, the mRNA levels of both CREB and PGC-1α were significantly reduced, indicating the involvement of ER stress in cisplatin-mediated PGC-1α expression regulation (Fig. 5c; Supplementary Fig. 4c).

To quantify the effects of cisplatin on PGC-1α transcriptional activity, HepAD38 cells were transfected with PGC-1α-luc reporter plasmid containing a luciferase ORF under the PGC-1α promoter. The data indicated that cisplatin treatment substantially induced PGC-1α promoter activity, whereas ER stress response inhibition by PBA or 4 μ8C significantly reduced cisplatin-provoked luciferase activity by approximately 70% (Fig. 5d). Moreover, chromatin immunoprecipitation assay revealed that ER stress inhibition significantly decreased CREB recruitment to the PCG-1α promoter (Fig. 5e; Supplementary Fig. 4d). These findings indicated that cisplatin upregulates PGC-1α expression via ER stress signal activation.

### Cisplatin-induced endogenous PGC-1α through ER stress supports HBV transcription

**Figure 6:**
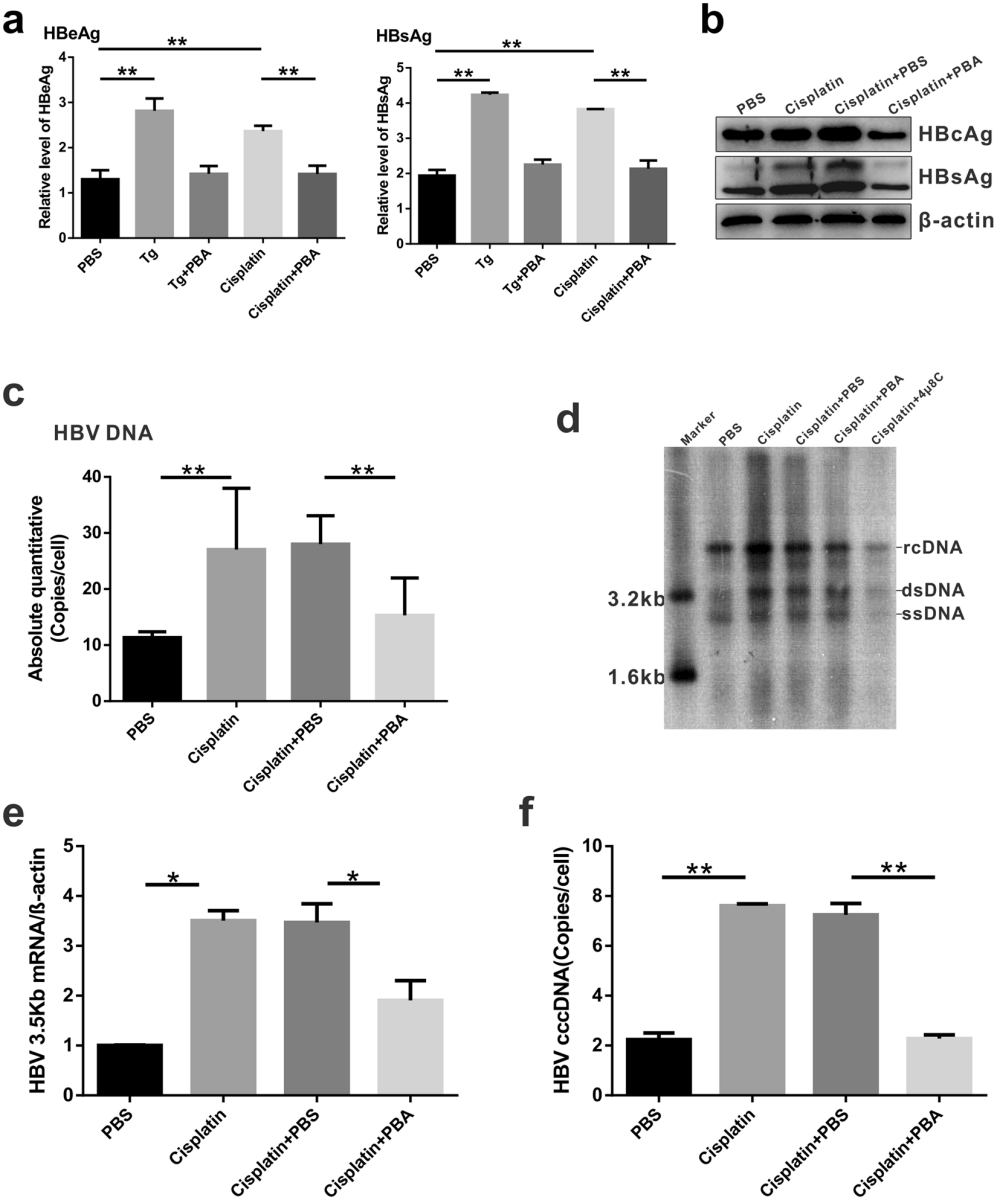
ER stress is required for cisplatin-induced HBV transcription. (**a**) HepAD38 cells were treated with cisplatin, thapsigargin, cisplatin combined with PBA or thapsigargin combined with PBA, respectively. The levels of HBsAg and HBeAg in the culture medium were measured by ELISA. (**b** to **c**) The expression of intracellular HBcAg and HBsAg (**b**), and HBV DNA (**c**) was determined by western blotting and quantitative PCR, respectively. (**d**) Southern blot analysis of intracellular HBV DNA. The cropped blots are used in the figure and full length blots are presented in Supplementary Fig. S10. (**e** to **f**) The levels of intracellular HBV 3.5 kb mRNA (**e**) and cccDNA (**f**) were analyzed by quantitative PCR. Data are shown as mean $\pm$ SD (independent experiments, n = 3); *p < 0.05, **p < 0.01.

Whether cisplatin-induced PGC-1α upregulation and HBV activation are dependent on ER stress response was investigated. ER stress inhibition with PBA or 4 μ8C was found to substantially suppress cisplatin-enhanced HBV transcription and HBeAg and HBsAg production (Fig. 6a,b; Supplementary Fig. 5a,b). Moreover, treatment with thapsigargin (ER stress inducer) alone also enhanced HBV replication and transcription through induction of ER stress. However, ER stress inhibitor-PBA strongly blocked Tg-induced HBV activation (Fig. 6a). In addition, treatment with ER stress inhibitor combined with cisplatin, cccDNA levels, 3.5-kb mRNA levels, and HBV DNA intermediates in HepAD38 cells, and HepG2-HBV1.1 cells sharply decreased compared with those with cisplatin treatment only (Fig. 6c to f; Supplementary Fig. 5c–e).

**Figure 7:**
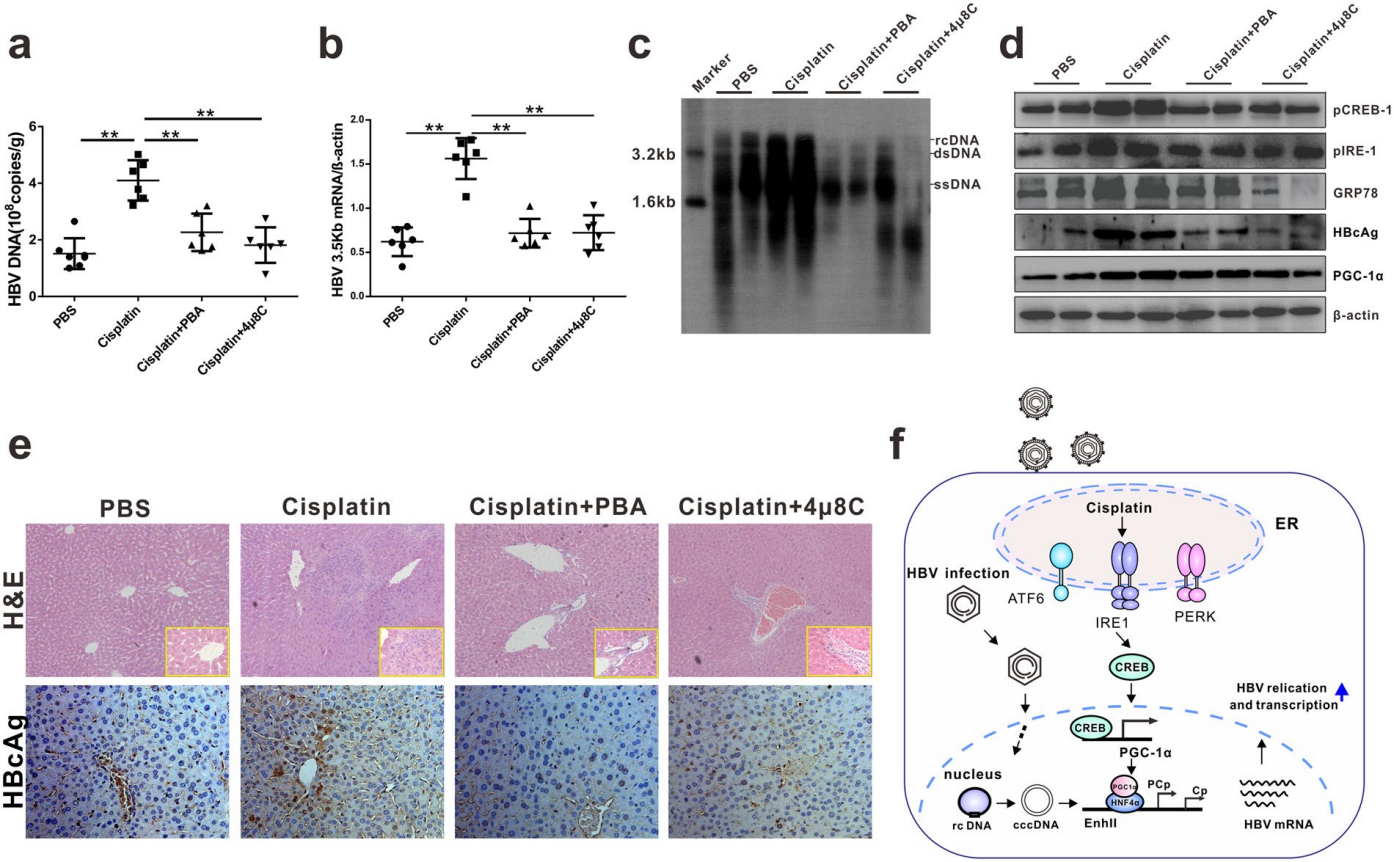
ER stress inhibitors alleviate cisplatin-induced HBV reactivation in HBV transgenic mouse model. HBV transgenic mice were randomly divided into four groups (n = 6 for each group). Animals received 100 mg/kg PBA or 10 mg/kg 4 μ8C for 1 day, followed by treatment with PBA or 4 μ8C combined with 6.0 mg/kg cisplatin daily for 5 days. On the sixth day, the mice were sacrificed and serum and tissue samples were collected. (**a** to **b**) HBV DNA and 3.5 kb mRNA in the liver were detected by real-time PCR. (**c**) HBV replication intermediates in the liver were detected by Southern blotting. (**d**) The expression of the indicated ER stress inducible proteins, HBcAg and PGC1α were analyzed by western blot. The cropped blots are used in the figure and full length blots are presented in Supplementary Fig. S11. (**e**) Liver tissue sections were H&E stained or stained with anti-HBc antibodies (magnification, ×200). (**f**) A schematic model by which cisplatin induces PGC1α through ER stress and strengthens HBV transcription.

Furthermore, whether HBV transcription is affected by ER stress inhibitor administration *in vivo* (Supplementary Fig. 6a) was validated. Cisplatin administration resulted in a significant HBV replication and HBsAg antigenemia. Treatment with ER stress inhibitor, PBA, or 4 μ8C via intraperitoneal administration substantially decreased serum HBeAg, HBsAg, HBV DNA, and ALT levels compared with cisplatin treatment alone (Supplementary Fig. 6b–e). Analysis of liver samples revealed that the induction of DNA replication, protein synthesis, and liver injury by cisplatin was remarkably reduced when ER stress inhibitor was co-administered. Moreover, combined treatment with ER stress inhibitor and cisplatin remarkably reversed cisplatin-induced expression of phosphorylated CREB-1, IRE1, and PGC-1α (Fig. 7a–e). These results suggested that cisplatin activates HBV replication possibly by HBV DNA-associated PGC-1α activation through ER stress response.

## Discussion

HBV reactivation is most commonly reported in patients undergoing anticancer therapy, immunosuppressive therapy, or organ and tissue transplantation. Recently, HBV reactivation has also been reported to occur in chronic hepatitis C (CHC) patients co-infected with HBV receiving direct-acting antiviral therapy^26,27^. Immune system reconstitution after the completion of chemotherapeutic or immunosuppressive therapy plays a central role in HBV reactivation and liver injury. However, the mechanism for the enhanced viral replication during cytotoxic chemotherapy remains unclear. In this study, we provide novel evidence that ER stress-PGC-1α signalling pathway is a critical regulator of cisplatin-induced HBV transcription and replication. Inhibition of the ER stress-PGC-1α pathway significantly blocked the stimulatory activity of cisplatin on HBV replication both in HBV-replicative hepatoma cells and in HBV-transgenic mouse model.

Our findings demonstrated that cisplatin strengthens HBV replication through upregulation of HNF-4α and PGC-1α expression. HBV is a double-stranded DNA virus that replicates by reverse transcription. Several transcription factors bound to the HBV core promoter are responsible for the transcription of HBV 3.5 kb mRNA, which plays a pivotal role in the HBV life cycle. Previous studies reported that doxorubicin and other chemotherapeutic medications enhanced HBV replication in HBV-expressing hepatoma cells^18^. Doxorubicin induced P21(Waf1/Cip1) expression and enhanced the binding of CCAAT/enhancer-binding protein α to the HBV core promoter, thereby contributing to enhanced HBV replication^28^ Recently, Mouler Rechtman M *et al*. reported that anticancer cytotoxic agents induce PGC-1α upregulation and coactivate HBV transcription^29^. Our results are consistent with previous findings that chemotherapeutic agents directly stimulate HBV replication through activation of the transcriptional factors HNF-4α and PGC-1α.

Potential HBV reactivation is associated with the persistence in the nucleus of infected hepatocytes of a stable HBV cccDNA^30,31^, which serves as the template for all HBV mRNA syntheses. High levels of post-transcriptional modification similar to chromosomal DNA results in active transcription of HBV DNA. HBx protein is reported to activate HBV transcription by recruitment of p300 and CBP acetyltransferase onto cccDNA^32^. Recently, protein arginine methyltransferase 5 induced epigenetic suppression of cccDNA transcription and interfered with pgRNA encapsidation^33^. Moreover, interferon-Iα or interleukin-6 treatment reduced active epigenetic modification of cccDNA and suppressed HBV transcription^34,35^. Our study showed that, besides HBV transcription level, both HBV cccDNA and 3.5-kb mRNA levels were enhanced by cisplatin. Liver-enriched transcription factors, such as HNF4 and retinoid X receptor α (RXRα), are capable of binding to cccDNA and contribute to the efficient cccDNA transcription^36^. Future studies are necessary to explore whether host factors and epigenetic regulators are involved in cisplatin-induced upregulation of cccDNA and 3.5 kb mRNA levels.

Another key finding of this study is that cisplatin could increase HBV core promoter activity and virus replication via enhancement of the metabolism regulator PGC-1α. Previous studies indicated that PGC-1α is a critical regulator in both HBV biosynthesis and gluconeogenesis^37,38^. An early study showed that PGC-1α coactivates HNF-4α to enhance HBV replication through nutritional signals^39^. PGC-1α activation by CREB is a common pattern in liver gluconeogenesis. Our results demonstrated that cisplatin induces CREB-PGC-1α activation and enhances HBV replication, indicating that HBV hijacks the energetic pathway for its production under certain environmental stress. HBV may tune in virus biosynthesis and protein production adaptive to external metabolic signal or physiological stress^40^. These data, combined with our results, suggest that the regulation of viral replication and metabolism might adopt similar hepatic signal transduction pathways. Interestingly, our results also demonstrated that other transcription factors such as CREB and RXRα were upregulated after cisplatin treatment. Except for an activator for PGC-1α transcription, CREB may bind to HBV core promoter and directly stimulate viral transcription. We speculated that other factors aside from ER stress and PGC-1α upregulation may also be involved in cisplatin-mediated HBV activation, which remains to be further studied. Another limitation of this study is that the replication status of HBV in our experiment model is distinct from that in clinical patients. In an HBV-replicating cell model or HBV mouse model, the replication levels of HBV are relatively high, but viral replication is negative or very low in clinical patients before HBV reactivation. Thus, cisplatin-induced HBV activation needs to be confirmed in more relevant models or in patients.

Importantly, we propose a model that illustrates the molecular mechanism underlying cisplatin-induced HBV activation. In this model, cisplatin upregulates PCG-1α through an ER stress-mediated, phosphorylated CREB-dependent pathway. Cisplatin induces ER stress and adaptive UPR activation in HBV replicative cells. IRE1, an arm of the UPR pathway, activates CREB-1 phosphorylation and upregulates PGC-1α expression. As an HBV transcription coactivator, PGC-1α indirectly binds to the HBV core promoter region through interaction with HNF4α. Lastly, inhibition of ER stress, IRE1 pathway, or PGC-1α expression rendered cisplatin-induced HBV biosynthesis *in vitro* and *in vivo* (Fig. 7f). The therapeutic potential of PGC-1α modulators or ER stress inhibitors have been demonstrated in an animal model^41^; hence, novel potential therapeutic strategies should target PGC-1α or ER stress for the treatment of chemotherapy-related HBV reactivation.

## Methods

### Cell cultures, transfection, *in vitro* viral infection, and chemotherapy

The stable HBV-expressing cell lines HepG2-HBV1.1 and HepAD38 cells were cultured and transfected using Lipofectamine^TM^ 2000 (Invitrogen, Carlsbad, CA, USA) as previously described^42^. HepG2-HBV1.1 cells were maintained in Dulbecco’s modified Eagle medium (DMEM, HyClone, Logan, UT, USA) supplemented with 10% fetal bovine serum (FBS, Gibco, Rockville, MD, USA), 100 units/mL penicillin, and 100 μg/mL streptomycin (HyClone). HepAD38 cells were cultured in DMEM/F-12 medium containing 400 ng/ml tetracycline to suppress HBV pgRNA transcription. At 7 days after tetracycline removal, HepAD38 cells were treated with chemotherapeutic agents.

HepG2 cells stably expressing sodium taurocholate co-transporting polypeptide (HepG2-NTCP) were infected with HBV virus as described previously^43,44^. Briefly, the cell culture medium of HepAD38 cells was filtered through a 0.45-μm filter and precipitated with 8% PEG8000 overnight at 4 °C. After precipitation, the concentrated HBV particles were used to infect HepG2-NTCP cells in the presence of 4% PEG8000 for 24 h.

For chemotherapy treatment, cells were incubated with 6 μM cisplatin (Nanjing Pharmaceutical Factory Co., Ltd., Jiangsu, China), 0.3 μM vincristine (Guangdong Lingnan Pharmaceutical Co., Ltd., Guangdong, China), or 0.6 μM epirubicin (Hisun Chemical Co., Ltd., Zhejiang, China) for 96 h. The medium was replaced with fresh serum-free DMEM medium for an additional 48-h incubation, and the cells were collected for detection. For ER stress induction, cells were treated with 100 nM ER stress inducer thapsigargin for 8 h and subsequently collected for analysis.

### Antibodies and reagents

The following primary antibodies were used for the western blot analysis: anti-PGC-1α (no. sc-13067), anti-HNF-4α (no. sc-374229), and anti-pCREB-1 antibodies (no. sc-81486), which were purchased from Santa Cruz Biotechnology (Santa Cruz, CA, USA). Antibodies to GRP78/BiP (no. ab32618), phosphor-IRE1(no. ab48187), and CREB-1 (no. ab32515) were obtained from Abcam (Cambridge, UK). Antibodies to IRE1α (no. 3294 P) and PERK (5683 P) were obtained from Cell Signaling Technology (Danvers, MA, USA). Anti-HBcAg (no. B0586), anti-HBsAg (no. NB100–62652), and anti-XBP-1s (no. 647501) antibodies were obtained from Dako (Dako, Glostrup, Denmark), Novus Biological (Littleton, CO, USA), and BioLegend (San Diego, CA, USA), respectively. IRE1 inhibitor 4-methylumbelliferone 8-carbaldehyde (4 μ8C, no. S7272) was obtained from Selleck Chemicals (Houston, TX, USA). 4-Phenylbutyric acid (PBA, no. P21005), thapsigargin (Tg, no. T9033), and lamivudine (LMV, no. Y0000426) were obtained from Sigma-Aldrich (St Louis, USA). Entecavir (ETV, no. 003) was obtained from Yingxuan Chempharm Co., LTD (Shanghai, China).

### Adenoviruses and reporter plasmids

Oligonucleotides containing siRNA target sites for the coding region of human or mouse HNF-4α and PGC-1α (Supporting Table S1) were annealed and subcloned into the Sfi I site of pSES vector (from Dr. T-C He, University of Chicago). The shuttle vectors were used to generate adenovirus AdR-siSnail using the AdEasy system^45^. The resultant adenoviral vector also expresses monomeric red fluorescent protein (RFP).

An ER stress reporter plasmid that contains a common motif of upstream ER stress response elements (pERSE-luc) was purchased from Beyotime Institute of Biotechnology, Shanghai, China^46^. The PGC-1α promoter-luciferase reporter was generated by cloning ~1 kb polymerase chain reaction (PCR) fragment into the pGL3-basic vector (Promega, Madison, WI, USA; #E1751).

### Primary hepatocyte isolation

Primary mouse hepatocytes were isolated by a conventional two-step collagenase perfusion method as described previously^47^.

### Cell Viability Assays

Cell viability was assessed by trypan blue staining assay. Cells were seeded in 24-well plates followed by exposure to medium containing phosphate-buffered saline (PBS) or the indicated concentration of cisplatin for 5 days. For trypan blue staining, adherent cells were collected and stained with 0.4% trypan blue (Beyotime, Nantong, China) for 5 min at 37 °C. The number of cells excluding trypan blue was counted in a hemocytometer and expressed as a percentage of viable cells compared with PBS-treated cells.

### Western blot analysis

Whole protein lysates were separated by 10% sodium dodecyl sulfate polyacrylamide gel electrophoresis and electrotransferred to polyvinylidene difluoride membranes (Millipore, Billerica, MA, USA). The blots were probed with the indicated antibodies. The secondary antibodies coupled to horseradish peroxidase were purchased from Abcam (Cambridge, UK). Protein bands were visualized with SuperSignal West Pico Chemiluminescent Substrate Kits (Millipore).

### Animal models

Hydrodynamic injection (HI) experiments were performed as described previously^48^. C57BL/6 J mice (6-8 weeks old, 6 mice per group) were provided by the Laboratory Animal Center of Chongqing Medical University (SCXK (YU) 2012-0001). Mice were treated daily with 6.0 mg/kg cisplatin or phosphate-buffered saline (PBS) control via tail vein injection for 5 days, after HI of HBV replication-competent plasmid pHBV1.3. For siRNA experiments, 10 μg of pHBV1.3 and AdR-siPGC-1α, AdR-siHNF-4α, or AdR-sicontrol were co-administered into the mice.

HBV-transgenic (HBV-Tg) mice (n = 6 for each group, kindly provided by Prof. Ning-shao Xia from the School of Public Health, Xiamen University)^49^ were intraperitoneally injected with 10.0 mg/kg 4-methylumbelliferone 8-carbaldehyde (4 μ8c),100 mg/kg phenylbutyric acid (PBA), or PBS control for 1 day, followed by treatment with 6.0 mg/kg cisplatin daily for five days. At the sixth day post-injection, the mouse serum and liver tissue samples were evaluated.

### Ethics Statement

The protocol for the use and care of animals was approved by the Institutional Animal Care and Use Committee at Chongqing Medical University (the project license number: 2014051). All mice were maintained under specific pathogen-free conditions in the laboratory animal center of Chongqing Medical University. Animal care and use protocols adhere to National Regulations for the administration of laboratory animal.

### Quantification of HBV DNA and cccDNA level by real-time polymerase chain reaction

Viral DNAs were precipitated with 35% PEG8000 and isolated from cytoplasmic core particle preparations by proteinase K digestion followed by phenol/chloroform extraction as described previously^31^. For cccDNA detection, the DNA samples were first pretreated with Plasmid-Safe DNase (Epicenter, Madison, WI). The extracted nuclear DNA was then analysed for cccDNA using TaqMan probe by real-time PCR with the indicated primers (Supplementary Table S1). The precipitated core particle DNA was measured by quantitative PCR or Southern blot. The plasmid pCH9/3091(containing 1.1 copies of HBV genome) served as a template for the standard curve.

### Southern blot analysis

The total HBV DNA was extracted from hepatoma cells or liver samples using the Wizard® Genomic DNA Purification Kit (Promega) and analysed by Southern blotting using a digoxigenin-labelled HBV-specific probe, as described previously^42^.

### Detection of HBV antigen, HBV DNA, and alanine aminotransferase in the mouse sera

Serum HBsAg and HBcAg levels in the mouse serum were determined with the commercially available radioimmunoassay kits (Beijing North Biotechnology Research Institute, Beijing, China). HBV DNA levels were quantified by a real-time PCR using SYBR Green dye as described previously^42^. Serum ALT was assayed with ELISA Kit (Bogoo Bio-Tech Co. Ltd, Shanghai, China), according to the manufacturer’s instructions.

### Immunohistochemistry

Paraffin-embedded liver tissue sections were stained with mouse anti-HBsAg antibody (ab20402, Abcam) or rabbit anti-HBcAg antibody (B0586, DAKO). Subsequently, tissue sections were incubated with Envision System and visualized using DAB substrate (Maixin-Bio, Fuzhou, China), according to the manufacturer’s instructions. IHC staining was quantitatively analysed using Image Pro Plus 6.0 software (Media Cybernetics, Inc., Rockville, MD, USA).

### RNA isolation and real-time PCR

Total RNA was isolated using TRIzol (Invitrogen, Rockville, MD) following the manufacturer’s instructions. cRNA served as a template to prepare a second round of cDNA using Moloney murine leukaemia virus reverse transcriptase (Promega). SYBR Green-based quantitative PCR analysis was performed using iQ5 real-time PCR detection system (Bio-Rad, CA, USA). HBV 3.5 kb mRNA quantification is normalized to genomic beta-actin. The primer sequences used for PCR are listed in Supplementary Table S1.

### Chromatin immunoprecipitation analysis

Chromatin immunoprecipitation analysis was performed as described previously^45^. Briefly, sonicated cell lysates were subjected to immunoprecipitation using 5 µg of the respective primary antibody (normal IgG served as control). Following elution, DNA was extracted and subjected to PCR analysis. Primers specific for the human *PPARGC1A* (PGC1a) promoters or HBV Enh II region (nt1650-nt1775) were used for PCR amplification (S1 Table). For chromatin immunoprecipitation-quantitative PCR, the immunoprecipitated DNA was quantitated by real-time PCR. Transcription factor enrichment was quantitated relative to the input control.

### Statistical analysis

All values were presented as means ± standard deviation (SD). Statistical significance was determined using one-way analysis of variance for multiple comparisons, and Student’s t-test was used to compare two groups. Probability values (p) <0.05 were considered statistically significant.

### Data Availability

All data generated or analysed during this study are included in this published article (and its Supplementary Information files).

## Electronic supplementary material

Supplementary Information

Supplementary Dataset 1-primer sequences
