# Supplementary material for: Cisplatin Enhances Hepatitis B Virus Replication and PGC-1α Expression through Endoplasmic Reticulum Stress
Source: Sci Rep. 2018 Feb 22;8:3496. doi: 10.1038/s41598-018-21847-3 (PMC5823916; doi:10.1038/s41598-018-21847-3)
Supplement: Supplementary file 1 — Supplementary Information [file 41598_2018_21847_MOESM1_ESM.pdf]

Supplementary Information

Cisplatin Enhances Hepatitis B Virus Replication and PGC-1α Expression through Endoplasmic Reticulum Stress

Xiaosong Li[1#], E Pan[1#], Junke Zhu[1#], Lei Xu [1#], Xuemei Chen[1], Jingjing Li[1], Li Liang[1], Yuan Hu[1], Jie Xia[1], Juan Chen[1], Wannan Chen[2], Jieli Hu[1], Kai Wang[1], Ni Tang[1,*], Ailong Huang[1,3*]

Affiliations

[1]Key Laboratory of Molecular Biology for Infectious Diseases (Ministry of Education), Institute for Viral Hepatitis, Department of Infectious Diseases, The Second Affiliated Hospital, Chongqing Medical University, Chongqing, China

[2]Key Laboratory of Tumor Microbiology, Fujian Medical University, Fuzhou Fujian, China.

[3]The Collaborative Innovation Center for Diagnosis and Treatment of Infectious Diseases (CCID), Zhejiang University, Hangzhou, China

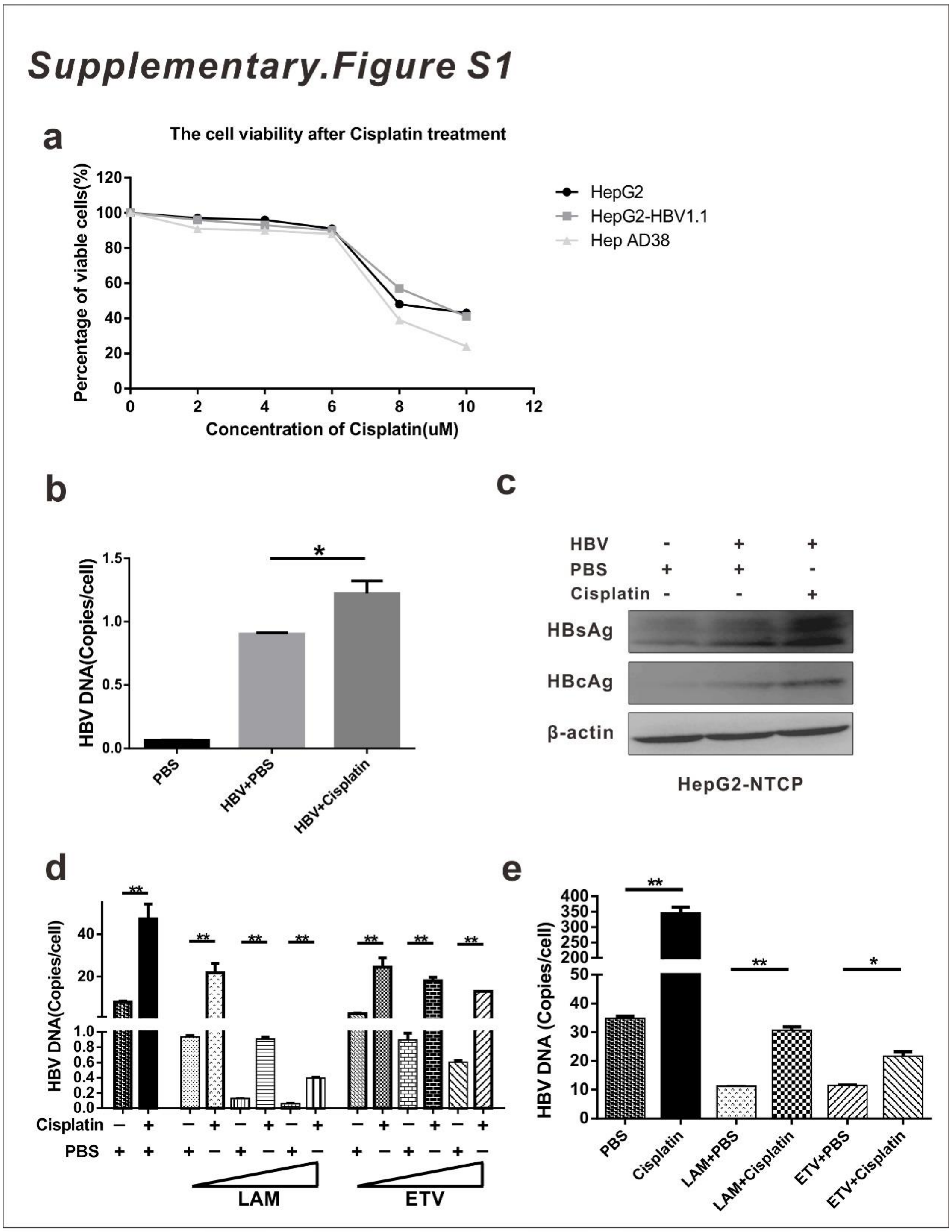

Supplementary Fig. 1. Cisplatin enhances HBV replication in HBV-expressing hepatoma cells. (**a**) Cell viability in HepAD38 cells treated with cisplatin. (**b**) Quantification of HBV DNA levels in HepG2-NTCP cells treated with cisplatin or PBS control. HBV-infected HepG2-NTCP Cells were treated with 6 μM cisplatin, and intracellular HBV DNA levels were determined at day

5 after treatment. Data represent the means ± SD, *$p < 0.05$. (**c**) Intracellular HBcAg and HBsAg were determined by western blotting in HepG2-NTCP cells. β-actin was used as loading control. (**d-e**) HepG2-HBV1.1 (d) and HepAD38 (e) cells were pretreated with lamivudine or entecavir, followed by 6 μM cisplatin treatment for another 5 days. Cell lysates were harvested and HBV DNA levels were quantified by real-time PCR assay. Data represent the means ± SD (independent experiments, n=3); *$p < 0.05$, **$p < 0.01$.

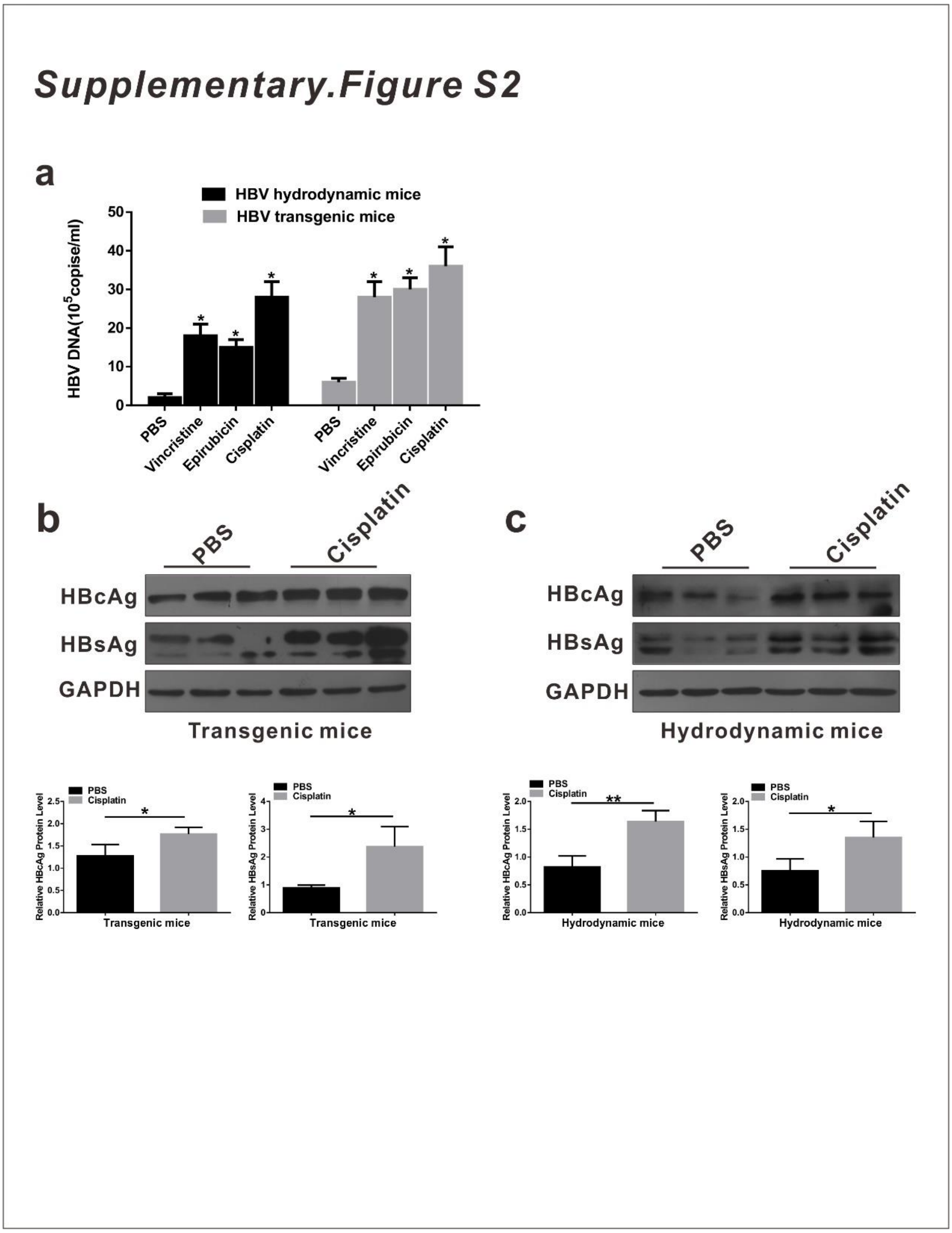

Supplementary Fig. 2. Cisplatin promotes HBV replication in HBV hydrodynamic and transgenic mouse model. (**a**) Serological markers of HBV DNA were assayed. (**b**) and (**c**) HBcAg and HBsAg in the liver were detected by western blotting. Three mice were analyzed per group, and at least three independent experiments were performed. GAPDH was used as

loading control. Integrated density was quantitatively analyzed using ImageJ software. *$p < 0.05$, **$p < 0.01$, Student's t-test.

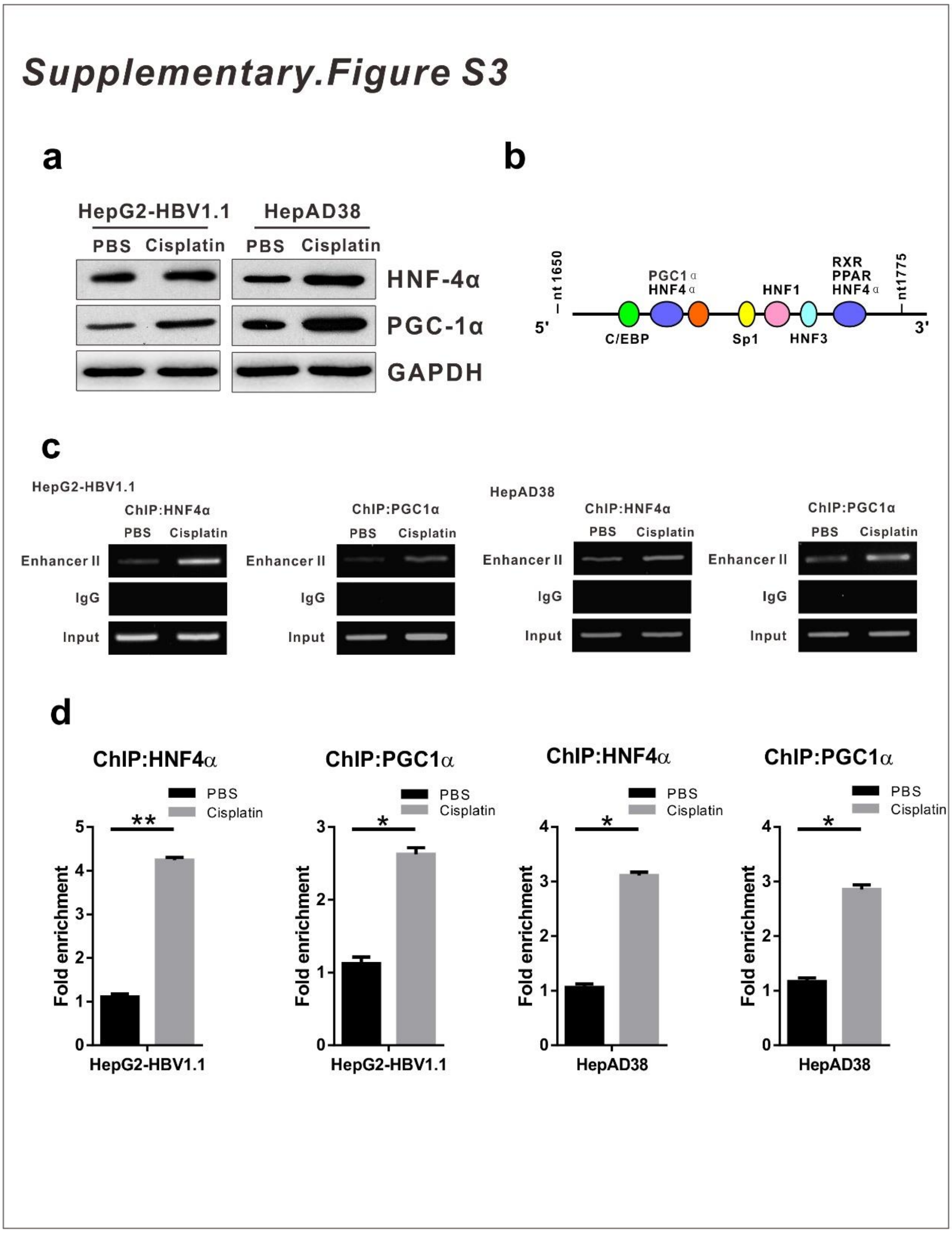

Supplementary Fig. 3. Cisplatin induces recruitment of HNF4α and PGC1α onto the Enhancer II region of HBV genome. (**a**) HepAD38 or HepG2-HBV1.1 cells were treated with cisplatin for 72 h. Cell lysates were analyzed by immunoblotting using anti-HNF4α and anti-PGC1α antibodies. Data are representative of three independent experiments. (**b**) Schematic

representation of transcription factor binding sites on the core promoter of the HBV genome. (**c**) HepAD38 and HepG2-HBV1.1 cells were treated with 6 μM cisplatin and used for chromatin immunoprecipitation assay with the indicated antibodies. The immunoprecipitated chromatin was amplified by the primers spanning the HNFα-binding site in the core promoter region of HBV genome. (**d**) Chromatin immunoprecipitation-quantitative PCR analysis. The relative fold enrichment (bound/input) was measured by quantitative PCR. *p<0.05, **p < 0.01.

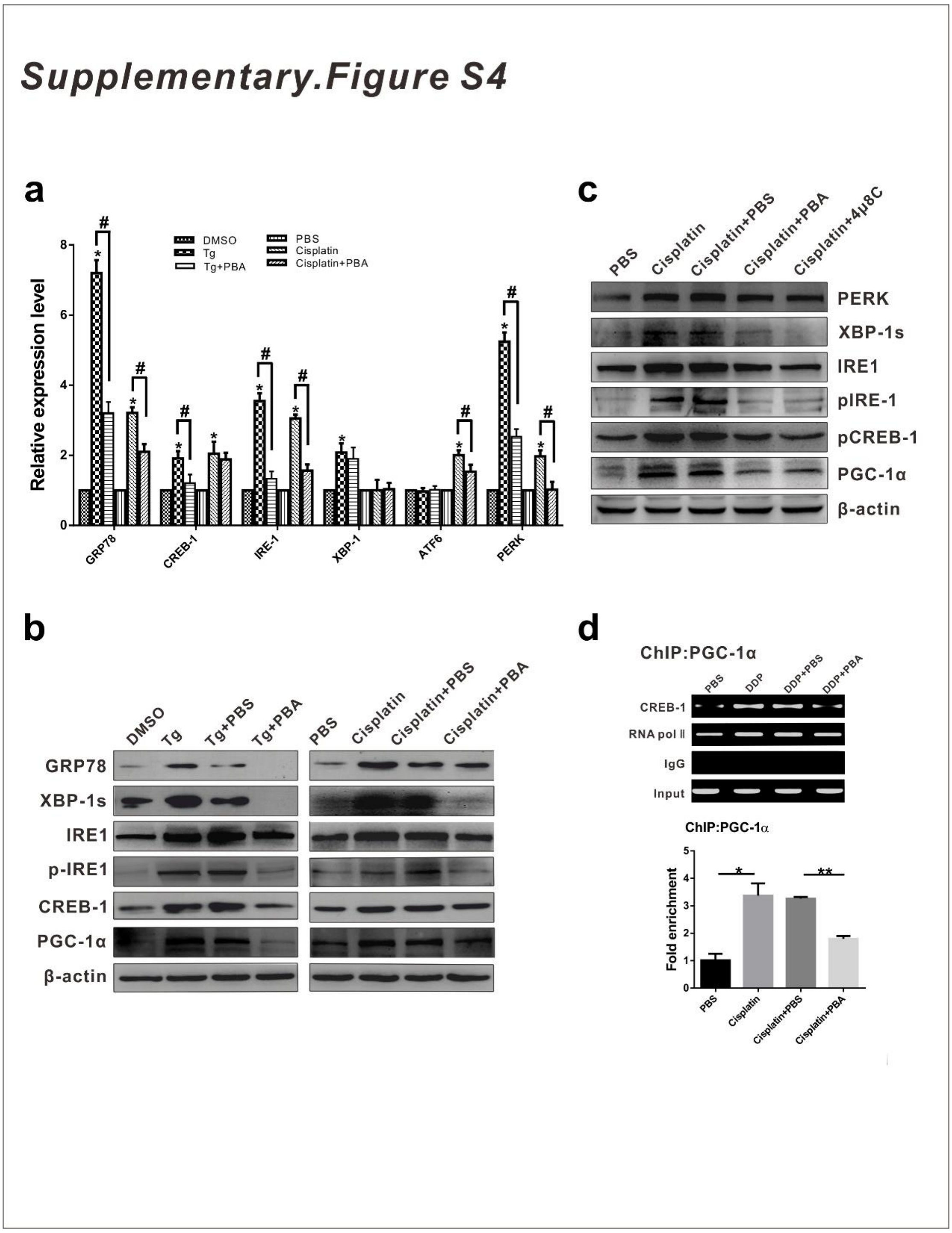

Supplementary Fig.4. Cisplatin-induced ER stress was alleviated by 4-PBA through regulation of several ER stress-inducible proteins. (**a**) HepG2-HBV1.1 cells were treated with cisplatin alone or cisplatin combined with ER stress inhibitor (4-PBA). The levels of the indicated ER inducible genes were determined by quantitative real-time PCR. Thapsigargin (Tg) is a

pharmacological inducer of ER stress. *$p < 0.05$, **$p < 0.01$ vs PBS control. $^{\#}p < 0.05$. (**b**) and (**c**) The protein levels of ER stress genes were analyzed by western blot in HepG2-HBV1.1 cells. (**d**) Chromatin immunoprecipitation assay of extracts from HepG2-HBV1.1 cells using CREB-1 antibody. Quantitative PCR results are shown as mean ± SD (independent experiments, n=3); *$p < 0.05$, **$p < 0.01$ by two-way analysis of variance.

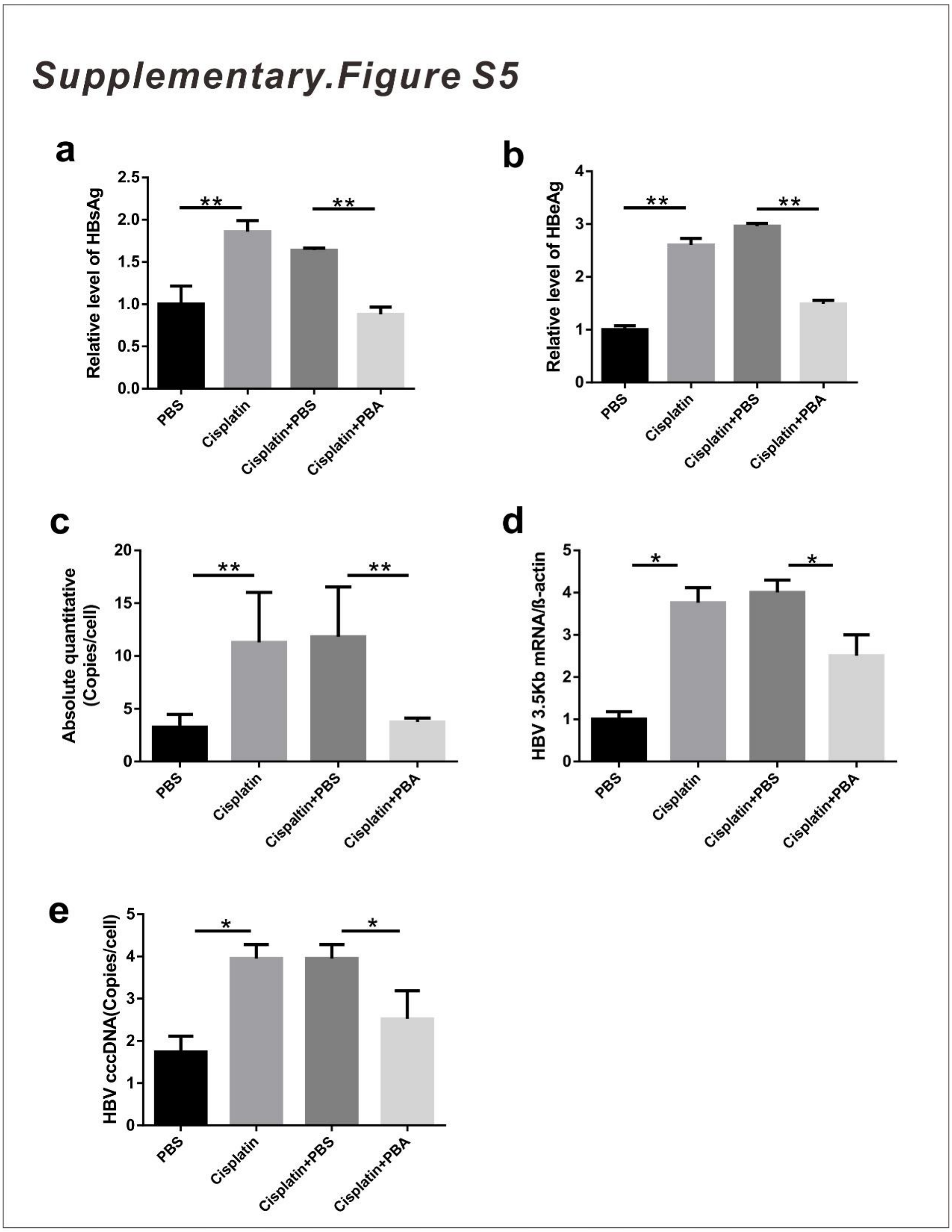

Supplementary Fig. 5. ER stress inhibitor 4-PBA alleviates cisplatin-induced HBV reactivation. HepG2-HBV1.1 cells were treated with cisplatin alone or cisplatin combined with ER stress inhibitor (2.46 μg/ml PBA) for 5 days. (**a to b**) The levels of HBsAg (a) and HBeAg (b) in the culture medium were measured by ELISA. (**c** t**o e**) The levels of intracellular HBV DNA (c),

3.5kb mRNA (d), and cccDNA (e) were analyzed by quantitative PCR. Data are shown as mean ± SD (independent experiments, n=3); *$p<0.05$, **$p < 0.01$ by two-way analysis of variance.

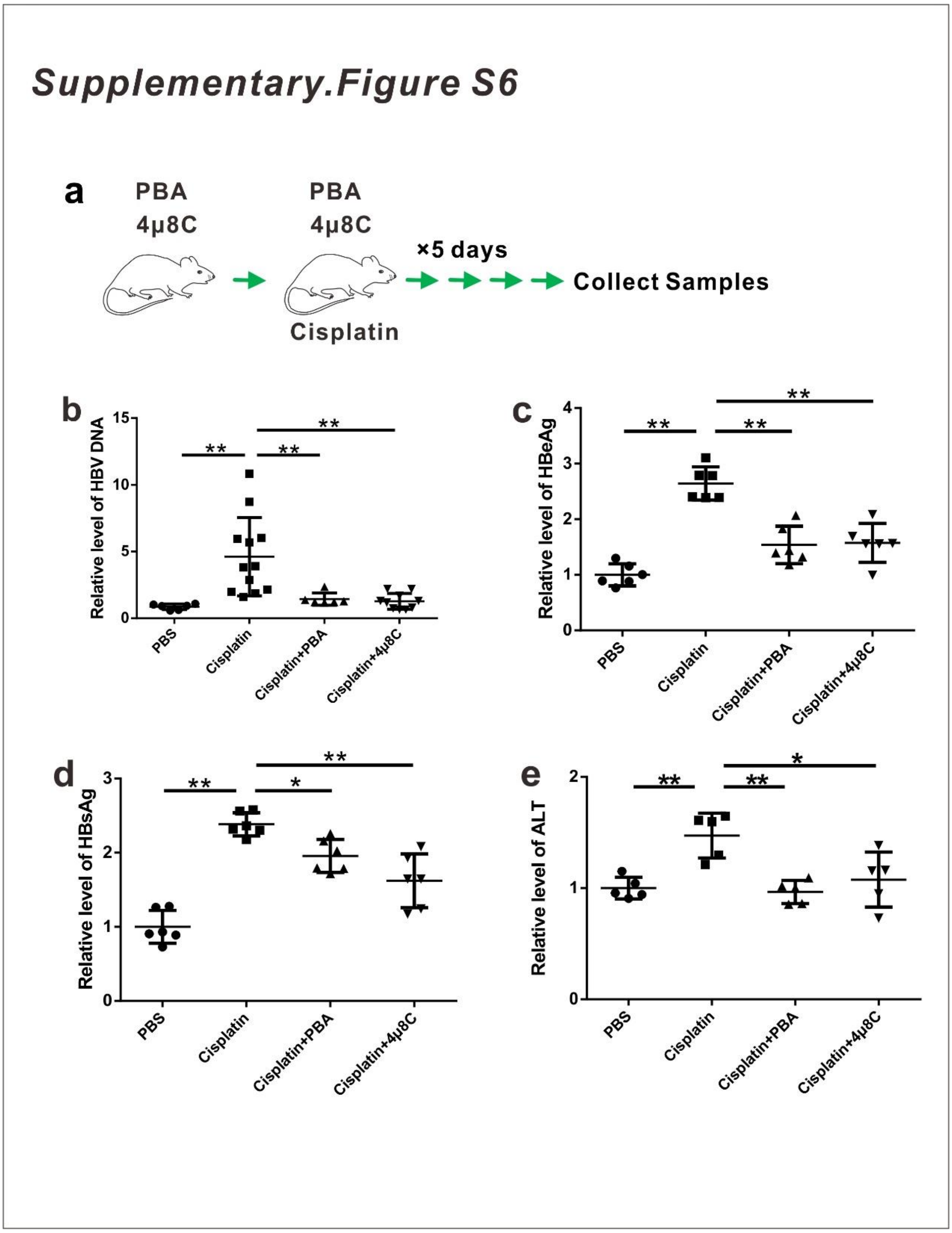

Supplementary Fig. 6. ER stress inhibitors abolished cisplatin-induced HBV reactivation in HBV-Tg mouse model. (**a**) Schematic representation of the experimental design. (**b to e**) Serological markers of HBV infection (HBV DNA, HBeAg, and HBsAg) and ALT were assayed. *p<0.05, **p < 0.01 by two-way analysis of variance.

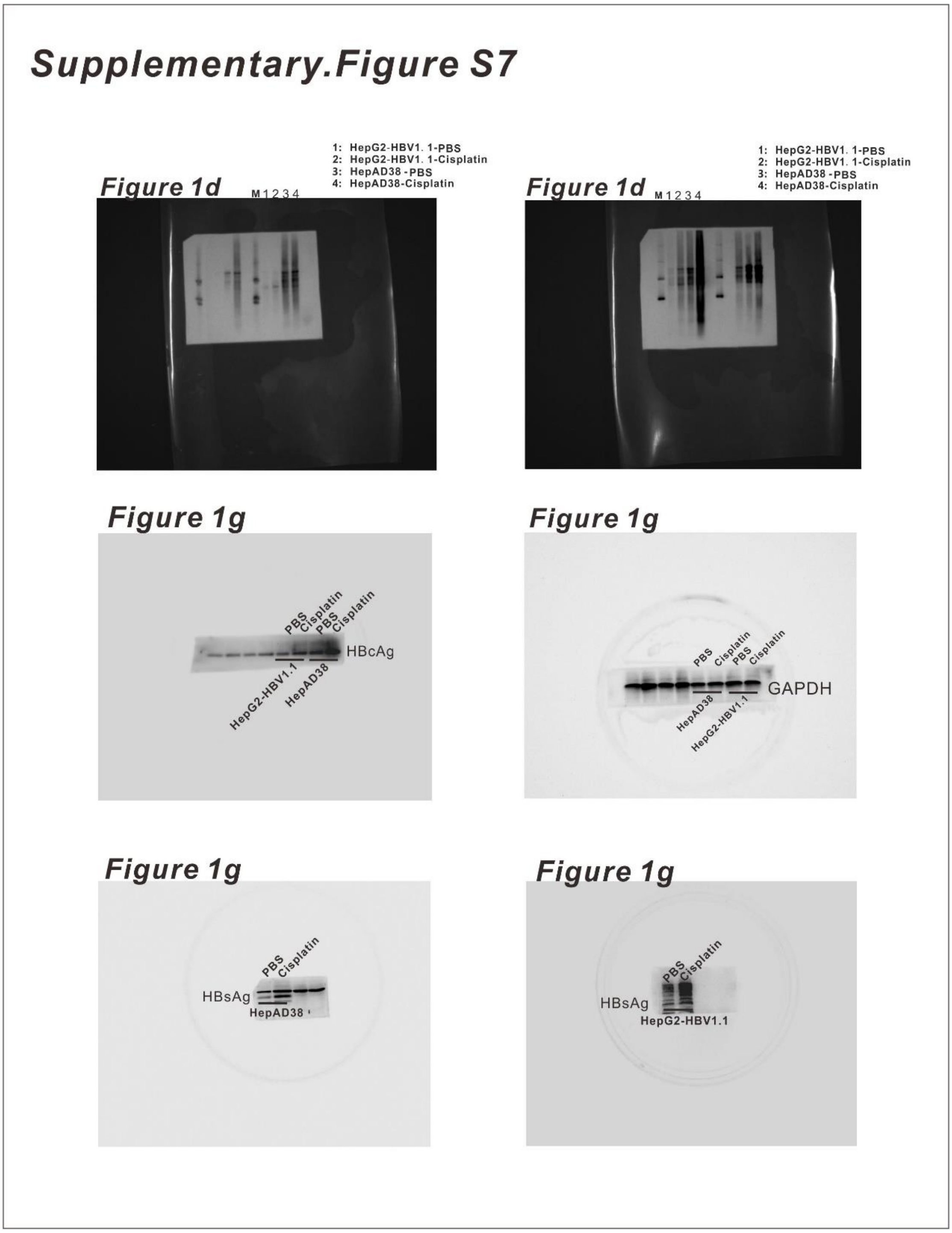

Supplementary Fig.7. Full-length blots of Figure 1.

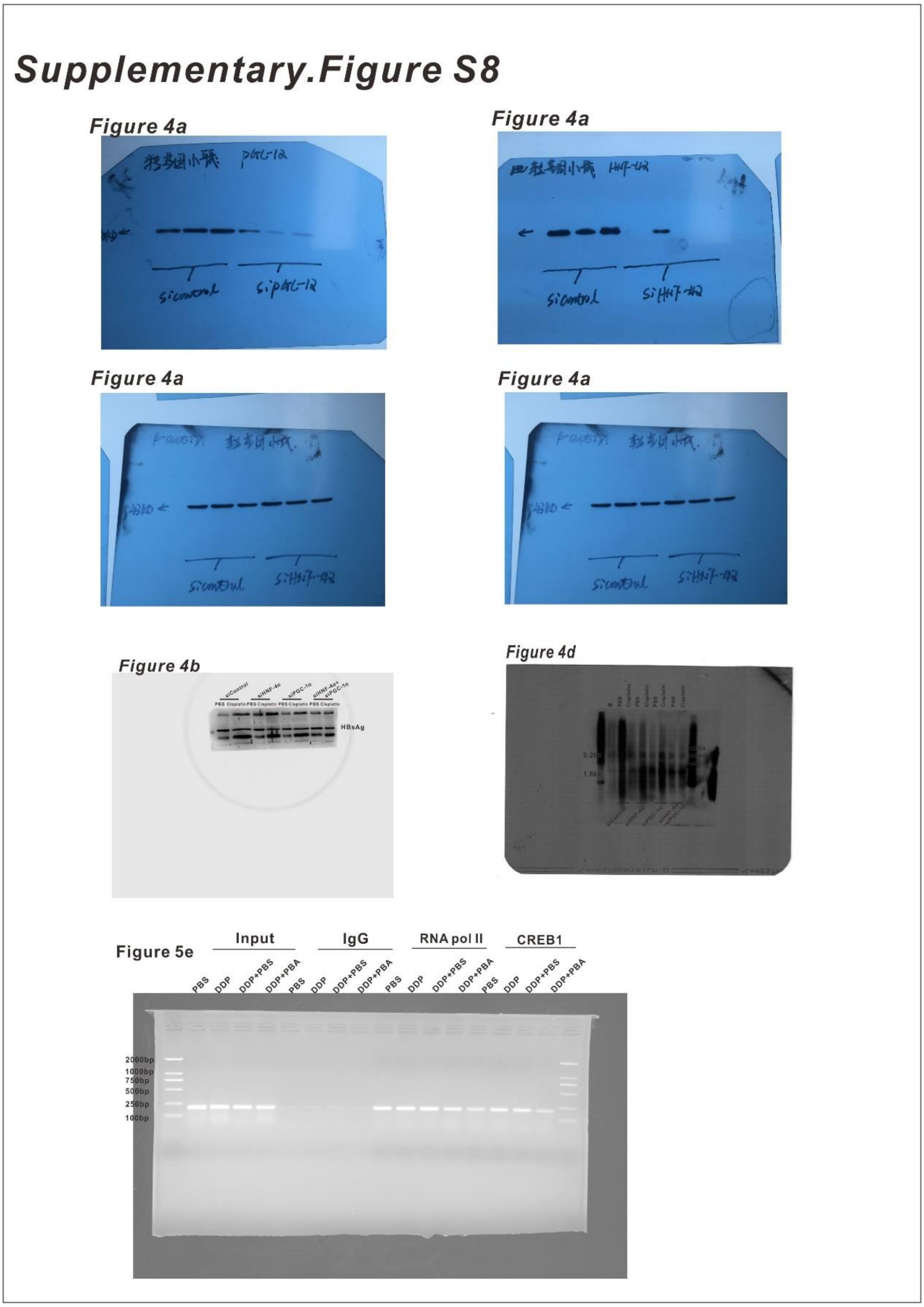

Supplementary Fig.8. Full-length blots of Figure 4 and Figure 5e.

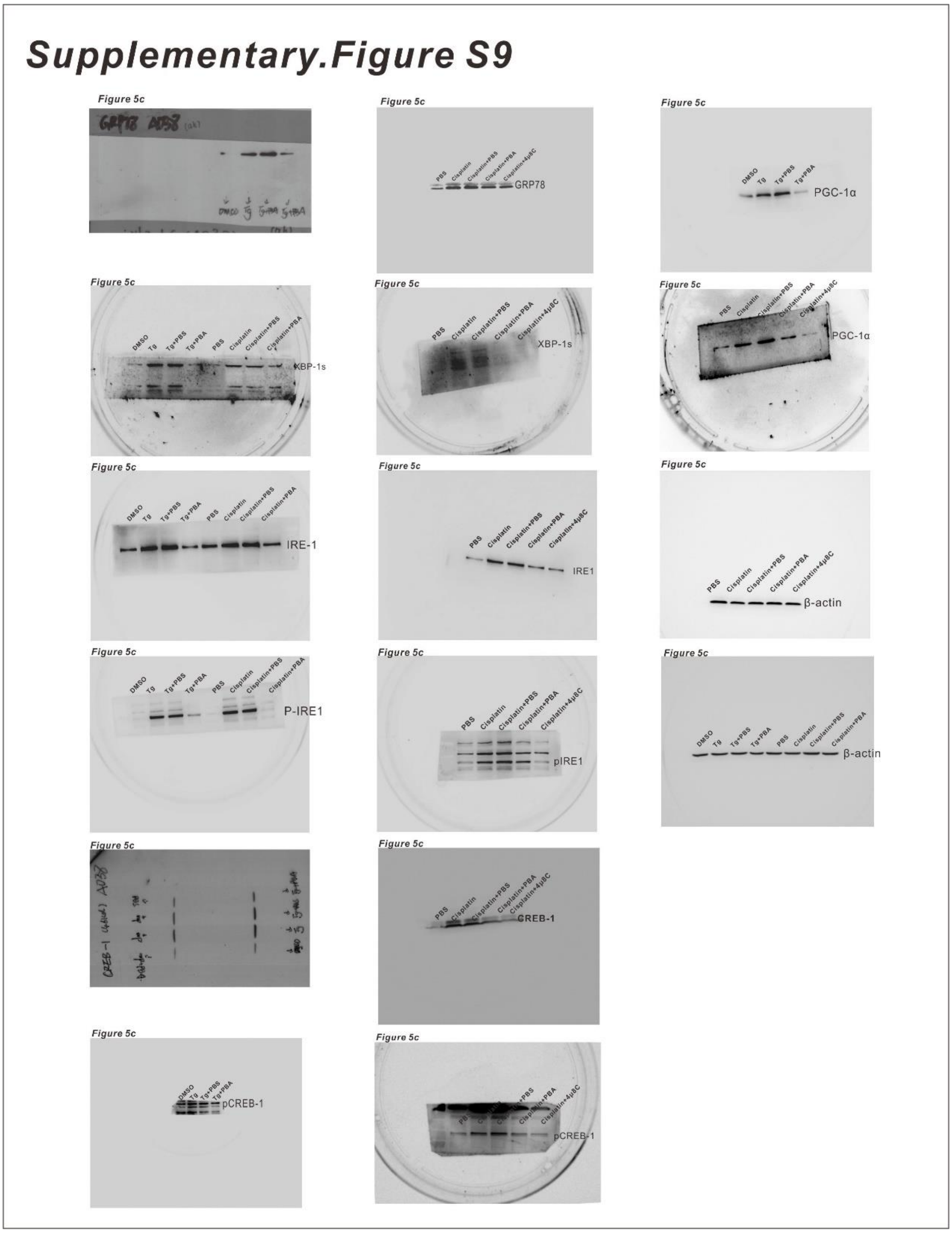

Supplementary Fig.9. Full-length blots of Figure 5.

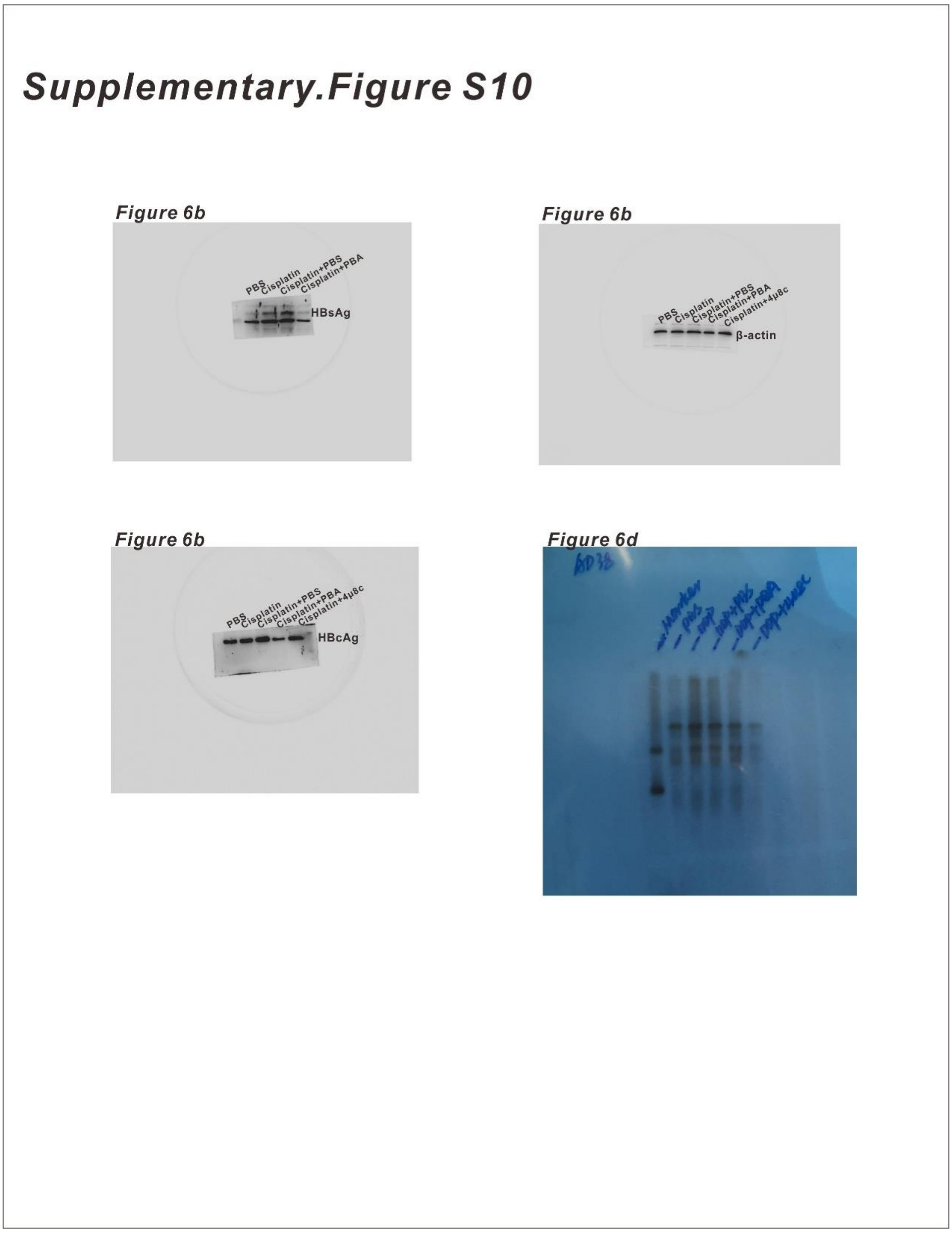

Supplementary Fig.10. Full-length blots of Figure 6.

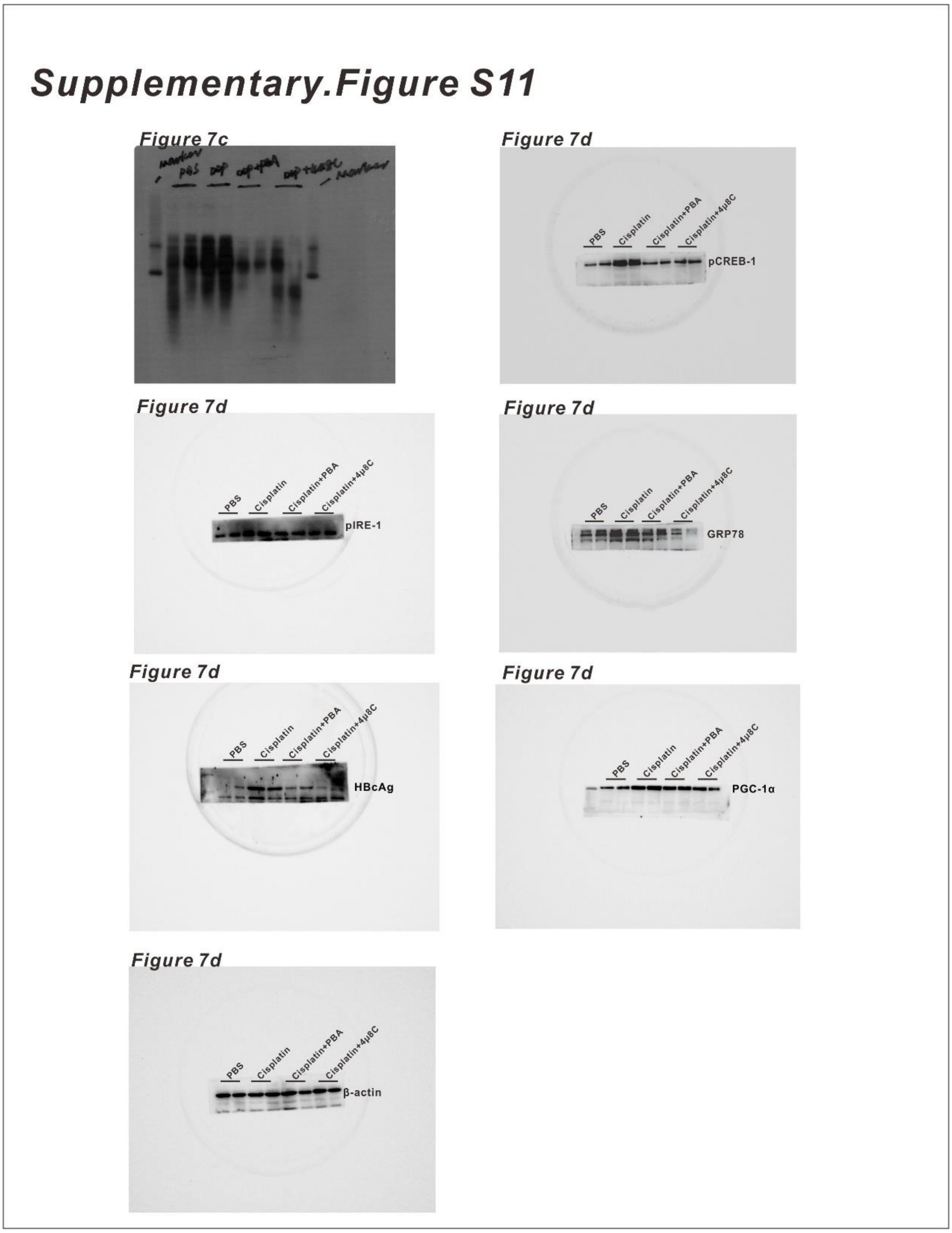

Supplementary Fig.11. Full-length blots of Figure 7.
